# VRK3 promotes KSHV infection by suppressing the antiviral type I interferon response

**DOI:** 10.1371/journal.ppat.1014400

**Published:** 2026-07-27

**Authors:** Caroline J. Yu, Blossom Damania

**Affiliations:** 1 Lineberger Cancer Comprehensive Center, University of North Carolina, Chapel Hill, North Carolina, United States of America; 2 Department of Microbiology & Immunology, University of North Carolina, Chapel Hill, North Carolina, United States of America; University of Wisconsin-Madison, UNITED STATES OF AMERICA

## Abstract

Kaposi sarcoma-associated herpesvirus (KSHV) is an oncogenic gammaherpesvirus that is associated with several human malignancies, and primary infection by KSHV can activate both DNA and RNA sensing pathways. Host innate immunity is a rapid, first-line defense against foreign pathogens, where pattern recognition receptors detect virus-associated antigens and activate the type I interferon response to prime the cell to an antiviral state to limit viral infections. However, viruses such as gammaherpesviruses, have evolved mechanisms to co-opt host factors to antagonize these pathways to promote persistent infection. The cellular vaccinia-related kinase (VRK) family is a trio of serine/threonine kinases, VRK1, VRK2 and VRK3, named for their homology to the vaccinia virus B1 kinase. The VRKs are involved in many cellular processes, including nuclear envelope assembly during mitosis and the DNA damage response. However, the role of the VRKs in KSHV infection and innate immunity has not previously been explored. We found that VRK3 is upregulated in response to KSHV primary infection of endothelial cells. VRK3 is also upregulated during KSHV reactivation from latency. Knockdown of VRK3 limited KSHV primary infection and lytic reactivation and resulted in increased activation of TBK1 and IRF3 and subsequent upregulation of interferon stimulated genes. Overexpression of VRK3 reversed this phenotype. Furthermore, we are also the first to demonstrate that VRK3 is a negative regulator of the interferon response even in the absence of viral infection. Collectively, our data highlight the importance of the host factor, VRK3, as a novel suppressor of the type I interferon response during the KSHV viral lifecycle.

## Author summary

Kaposi sarcoma-associated herpesvirus (KSHV) is the causative agent of Kaposi sarcoma and several lymphoproliferative disorders. KSHV persists as a lifelong infection by manipulating cellular factors to evade antiviral immunity. The innate immune system can detect and limit viral infections. Innate immune responses are tightly regulated by host factors to prevent excessive immunity. Here, we found that the cellular protein vaccinia-related kinase 3 (VRK3) limits antiviral signaling in KSHV infection. Our work identifies VRK3 as a previously unknown modulator of antiviral immunity and underscores how viruses, like KSHV, can exploit cellular immune control pathways to evade host defense. Understanding this interaction may help guide future strategies aimed at restoring antiviral immunity in virus-associated cancers.

## Introduction

Kaposi sarcoma-associated herpesvirus (KSHV) and Epstein-Barr Virus (EBV) are double-stranded DNA gammaherpesviruses associated with different human cancers. KSHV is the etiologic agent of the malignancies, Kaposi sarcoma and primary effusion lymphoma (PEL), as well as the lymphoproliferative disorder, multicentric Castleman disease. KSHV establishes lifelong infection in humans and has a two-phase lifecycle, latent and lytic. During latency, viral genomes are tethered to host chromatin and there is limited viral gene expression. Upon reactivation, KSHV enters its lytic phase with robust viral replication to produce infectious virions. KSHV encodes viral factors and co-opts host factors to evade immune detection and dysregulate cellular processes that support viral persistence [1–3].

The innate immune system is the first line of antiviral defense. Pattern recognition receptors (PRRs) are sensors of pathogen-associated molecular patterns (PAMPs) such as aberrant RNA or DNA in the cytoplasm. Gammaherpesviruses, including KSHV and EBV, are sensed by several PRRs including the Toll-like receptors, RIG-I, IFI16, MDA5, and cGAS. Upon detection, activated PRRs transduce a signaling cascade through adaptor proteins, kinases, and transcription factors, to produce type I interferons (IFN) to drive the expression of interferon stimulated genes. The antiviral response relies on interferon regulatory factor (IRF)-mediated production of type I interferons, as well as activation of nuclear factor-κB-mediated induction of inflammatory cytokines that broadly limit viral infection. Gammaherpesviruses have co-evolved with their hosts, and as a counter defense, they can evade these innate immune pathways [4].

The cellular vaccinia-related kinase (VRK) family is a group of serine/threonine protein kinases comprised of three members: VRK1, VRK2, and VRK3. The VRKs are homologous to one another and are named for their sequence similarity to the catalytic domain of the vaccinia virus B1 kinase (VACV B1) [5–7]. Sequence alignments and phylogenetic analyses indicate that VRK1 and VRK2 are more closely related with one another than VRK3 [5].

VRKs are widely expressed in a variety of cell types and play a regulatory role in many cell processes, including cell cycle control, DNA damage, and transcription. VRK1 is the most extensively characterized family member and is critical for cell proliferation, especially in rapidly dividing cells [8–10]. The VRK family members are implicated in multiple cancers including breast cancer, glioblastomas, and hepatocellular carcinomas [11–17].

VRK1 has also been implicated in promoting type I interferon responses [18]. While initially thought to be catalytically inactive, VRK3 has been shown to possess kinase activity and can also function as a scaffolding protein [10,19,20]. VRK3 negatively regulates the ERK pathway through its interaction with the vaccinia H1-related phosphatase (VHR) [21–25]. Both VRK1 and VRK3 contain nuclear localization signals and are found in the nucleus and cytosol, whereas the major isoform of VRK2 associates with the endoplasmic reticulum [5]. The VRK2A isoform localizes to the endoplasmic reticulum and outer mitochondrial membrane, where it modulates the intrinsic apoptotic pathway through its direct interaction with Bcl-xL and regulation of BAX gene expression [26]. Notably, VRK2 has been shown to play a role in EBV by interacting with EBV BHRF1, a viral homolog of cellular Bcl-2, to enhance cell survival by preventing apoptosis [27]. Despite some shared localization and overlapping phosphorylation targets, VRKs exhibit distinct functional roles. In cell cycle progression, VRK1 primarily affects the G1/S phase transition, while VRK3 affects S phase progression and G2/M phase entry and exit [28].

Given the role of VRK1 in promoting innate immunity and the established role of VRK2 in EBV infection, we examined the role of the VRKs in KSHV infection. Here we have identified a novel role for VRK3 as a suppressor of the type I interferon response both in the presence and absence of viral infection. Loss of VRK3 in endothelial cells enhanced activation of the TBK1-IRF3 signaling axis, a critical signaling junction in antiviral innate immunity. VRK3 mediated this effect through the innate immune sensor, RIG-I, where RIG-I knockdown rescued the VRK3 depletion phenotype on the type I interferon response. We also investigated the role of the VRK family in KSHV infection and found that VRK3 depletion inhibits both primary KSHV infection and lytic reactivation. Together, these findings establish VRK3 as a novel, cellular factor that limits the type I interferon response and contributes to KSHV infection.

## Results

### VRK3 promotes KSHV infection

**Fig 1:**
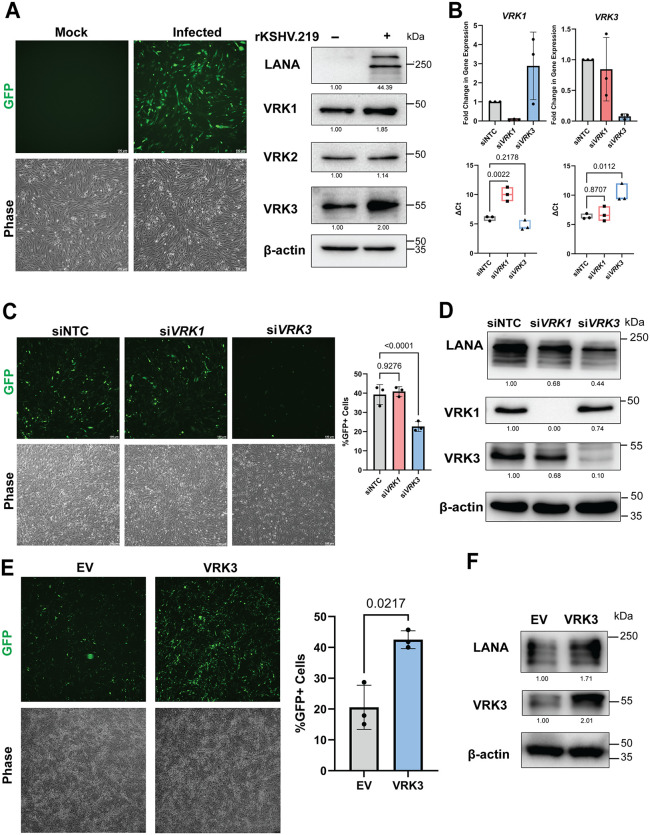
VRK3 promotes de novo KSHV infection. **(A)** HUVEC cells were infected with rKSHV.219 for 72 hours and then collected for analysis (n = 3). Cells were imaged by fluorescence microscopy to confirm KSHV infection by GFP signal (n = 3). The images shown are representative of images obtained from three biological replicate experiments. Mock- versus KSHV-infected HUVEC cell lysates collected at 72 hours post-infection were prepared and analyzed for VRK family protein expression by immunoblot with the indicated antibodies. The immunoblot shown here is representative of three biological replicate experiments. Quantification of the representative replicate is shown. However, quantification of all three biological replicates can be found in S3 Table. For Fig 1B-1D, HUVEC cells were transfected with non-targeting control (NTC), VRK1-, or VRK3*-* targeted pooled siRNA for 24 hours, then infected with rKSHV.219 for 72 hours (n = 3). **(B)** Cell pellets were collected for RNA isolation and RT-qPCR was subsequently performed to quantify *VRK1* and *VRK3* mRNA transcripts to validate knockdowns (n = 3). Fold change in gene expression is shown and p-values were analyzed by one-way ANOVA test from ΔCt values. **(C)** GFP^+^ cells were imaged by fluorescence microscopy at 72 hours post-infection (n = 3). GFP^+^ cells were also quantified by flow cytometry (n = 3). p-values were determined by a one-way ANOVA test and error bars indicate the standard error from the mean of three biological replicates. **(D)** Cell lysates were prepared and analyzed by immunoblot for KSHV LANA, VRK1, and VRK3 protein expression (n = 3). For Fig 1E-1F, HUVEC cells were transfected with either an empty vector or VRK3 expression plasmid for 48 hours, infected with rKSHV.219 for 72 hours and then collected for analysis (n = 3). **(E)** KSHV infection was measured by analyzing GFP signal using fluorescence microscopy (n = 3) and quantitating the percentage of GFP^+^ cells by flow cytometry (n = 3). p-values were determined from a student’s t-test and error bars indicate the standard error from the mean of three biological replicates. **(F)** At 72 hours post-infection, cells were harvested and lysed and analyzed for protein expression of KSHV LANA and cellular VRK3 (n = 3).

KSHV can infect a broad tropism of cells, including epithelial, endothelial, and B cells [29]. We infected immortalized hTERT-human umbilical vein endothelial cells (HUVEC) with the recombinant KSHV.219 virus (rKSHV.219), which encodes a green fluorescent protein (GFP) reporter (Fig 1A). At 72 hours post-infection, we observed that KSHV infection upregulates the protein expression of VRK1 and VRK3, but not VRK2 (Fig 1A). Notably, VRK3 exhibited the most pronounced increase in protein expression relative to VRK1 and VRK2.

To assess the roles of VRK1 and VRK3 during KSHV infection, HUVEC cells were transfected with pooled siRNAs targeting VRK1 and VRK3 transcripts, or non-targeting control (NTC) siRNA. Twenty-four hours post-transfection, cells were infected with rKSHV.219. At 72 hours post-infection, knockdowns were validated by RT-qPCR and immunoblot (Fig 1B and 1D). KSHV infection was confirmed by KSHV viral protein, LANA, by immunoblot (Fig 1D) and infection efficiency was evaluated by fluorescence microscopy and quantified by flow cytometry analysis of GFP-positive cells (Fig 1C). Knockdown of VRK1 did not significantly affect KSHV infection compared to the NTC condition. However, depletion of VRK3 resulted in a reduction of KSHV infection relative to NTC siRNA transfected cells, as seen by reduced GFP signal (Fig 1C). Pooled siRNA results were consistent with individual sequence-targeted siRNAs, where cells transfected with individual VRK3 siRNAs compared to cells transfected with NTC siRNA each also led to a significant decrease in KSHV infection (S1A and S1B Fig). Overall, this suggests that despite homology to one another, VRK1 and VRK3 have distinct roles, with VRK3 uniquely promoting de novo KSHV infection.

To further investigate the contribution of VRK3 to KSHV infection, we overexpressed VRK3 in HUVEC cells, infected with rKSHV.219, and examined the impact on KSHV infection. HUVEC cells were transfected with either an empty vector (EV) control or VRK3 expression plasmid, and then 48 hours later, were infected with rKSHV.219. Seventy two hours post-infection, KSHV infection was evaluated by both fluorescence microscopy and flow cytometry by GFP-positive cells (Fig 1E). By immunoblot, we confirmed KSHV infection, by probing for the KSHV viral protein, LANA, and VRK3 overexpression (Fig 1F). KSHV-infected cells with VRK3 overexpression reversed the phenotype observed in VRK3 depletion. Cells over-expressing VRK3 displayed increased KSHV infection compared to cells transfected with empty vector (EV), supporting the fact that VRK3 plays a pro-viral role in KSHV infection.

### VRK3 is required for optimal KSHV reactivation from latency

Given the contribution of VRK3 to KSHV primary infection, we wanted to investigate whether VRK3 also plays a role in KSHV lytic reactivation. KSHV displays two phases of its lifecycle: latency and lytic replication. During latency, the viral episome is tethered to host chromatin and replicated in sync with host cell division. During lytic replication, all viral genes are expressed and infectious virions are made [1].

**Fig 2:**
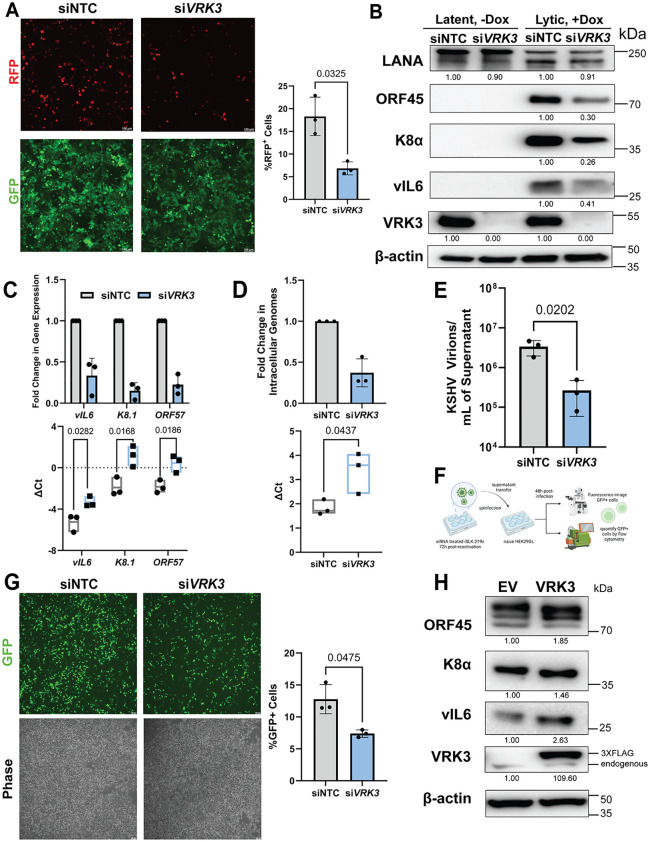
VRK3 contributes to KSHV reactivation. For Fig 2A-2G, latently-infected iSLK.219 cells were treated with non-targeting control (NTC) or VRK3*-*targeted pooled siRNA for 48 hours, then reactivated with 50 ng/mL doxycycline for 72 hours (n = 3). **(A)** At 72 hours post-reactivation, cells were imaged by fluorescence microscopy for RFP and GFP signal (n = 3), and the percentage of RFP^+^ cells relative to the non-targeting control were quantified by flow cytometry (n = 3). **(B)** Latent samples were collected prior to reactivation and compared to samples collected 72 hours post-reactivation. Cell lysates were prepared and analyzed by immunoblot with LANA, ORF45, K8α, and vIL6 to examine viral protein expression and VRK3 to confirm knockdown (n = 3). **(C)** At 72 hours post-reactivation, cell pellets were harvested for RNA extraction and subsequent cDNA synthesis, then RT-qPCR was performed to quantify viral transcripts, *vIL6*, *K8.1*, and *ORF57* (n = 3). Fold change in gene expression is shown and error bars indicate the standard error from the mean of three biological replicates. p-values were analyzed by multiple t-test from ΔCt values. **(D)** Intracellular genomes were quantified by real-time qPCR using KSHV ORF39 primers relative to β-actin primers (n = 3). Fold change in gene expression is shown and error bars indicate the standard error from the mean of three biological replicates. p-values were analyzed by a student’s t-test from ΔCt values. **(E)** Virions secreted in the supernatant were quantified relative to a standard curve of KSHV ORF39 plasmid (n = 3). p-values were determined by a student’s t-test and error bars indicate the standard error from the mean of three biological replicates. **(F)** At 72 hours post-reactivation, supernatants collected from NTC or VRK3 siRNA transfected iSLK.219 cells were transferred to naïve HEK293 cells. Cells were infected for 48 hours then analyzed for KSHV infection by GFP signal using fluorescence microscopy and GFP^+^ cells were quantified by flow cytometry in **(G)**. **(G)** KSHV infection from iSLK.219 supernatant transfer to HEK293 cells was analyzed by quantitating GFP^+^ cells by fluorescence microscopy and flow cytometry (n = 3). p-values were determined from a student’s t-test and error bars indicate the standard error from the mean of three biological replicates. **(H)** iSLK.219 cells were transfected with either an empty vector or VRK3 expression plasmid for 48 hours, then lytically induced with 50 ng/mL doxycycline treatment for 72 hours. At 72 hours post-reactivation, cell lysates were analyzed by immunoblot for expression of viral proteins, ORF45, K8α, and vIL6 and confirmation of VRK3 overexpression (n = 3).

We examined KSHV lytic reactivation using the KSHV-infected iSLK.219 cells, which constitutively express GFP and conditionally express RFP upon doxycycline-induced lytic reactivation driven by the KSHV polyadenylated nuclear RNA (PAN) promoter. These cells harbor latent KSHV and express the viral lytic transactivator, replication and transcription activator (RTA), under the control of a doxycycline-inducible promoter [30]. Consequently, treatment with doxycycline induces RTA expression and triggers viral reactivation [30]. We transfected iSLK.219 cells with either pooled siRNA targeting VRK3 transcripts or a non-targeting control (NTC) siRNA, and 48 hours later, induced reactivation with doxycycline treatment. After 72 hours of lytic reactivation, we examined the impact of VRK3 knockdown on KSHV reactivation. We examined lytic reactivation by RFP and GFP signals using fluorescence microscopy and quantitated RFP-positive cells by flow cytometry. Compared to the NTC-treated cells, VRK3-depleted cells had a decreased percentage of RFP-positive cells, indicating a lower level of lytic reactivation (Fig 2A). We then compared several KSHV viral proteins, LANA, ORF45, K8α, and vIL6, between latent and lytic cells (Fig 2B). As expected, only LANA was expressed to detectable levels during latency, and upon reactivation, lytic proteins were induced. We observed that ORF45, K8α, and vIL6 protein expression was reduced in VRK3-depleted cells relative to NTC-treated cells. At 72 hours post-lytic induction, we looked at a panel of KSHV mRNA transcripts, *vIL6*, *K8.1*, and *ORF57*, by RT-qPCR. When we compared cells transfected with VRK3 siRNA to cells transfected with NTC siRNA, we found a decrease in lytic viral transcripts (Fig 2C). Furthermore, after lytic induction, we quantitated intracellular viral DNA genomes, as well as viral genomes in the supernatant, and found a reduction of viral genomes in both compartments in cells transfected with VRK3 siRNA relative to NTC siRNA (Fig 2D and 2E). Additionally, 72 hours post-lytic reactivation we transferred both the NTC and VRK3 pooled siRNA-treated iSLK.219 supernatants to naïve HEK293 cells to confirm the infectivity of virions produced using fluorescence microscopy and flow cytometry (Fig 1F). We observed a noticeable decrease in GFP-positive cells with the VRK3-pooled siRNA-treated samples compared to the control NTC siRNA-treated samples (Fig 2G). Pooled siRNA results were consistent with individual sequence-targeted siRNAs, where cells transfected with individual VRK3 siRNAs compared to cells transfected with NTC siRNA also led to a significant decrease in lytic reactivation as measured by RFP-positive cells and viral protein expression (S1C and S1D Fig).

We also tested whether VRK3 overexpression increased KSHV reactivation. We transfected iSLK.219 cells with either an empty vector (EV) control or VRK3 expression plasmid, and then 48 hours later, induced lytic reactivation with doxycycline treatment. Seventy-two hours after reactivation, we examined the impact on KSHV lytic markers. VRK3 overexpression modestly increased lytic protein expression compared to the EV control (Fig 2H), further confirming that VRK3 contributes to KSHV lytic reactivation. Thus, it appears that during KSHV reactivation, VRK3 expression is required for optimal viral transcripts, viral proteins, and virion production.

### VRK3 promotes KSHV reactivation in PEL

**Fig 3:**
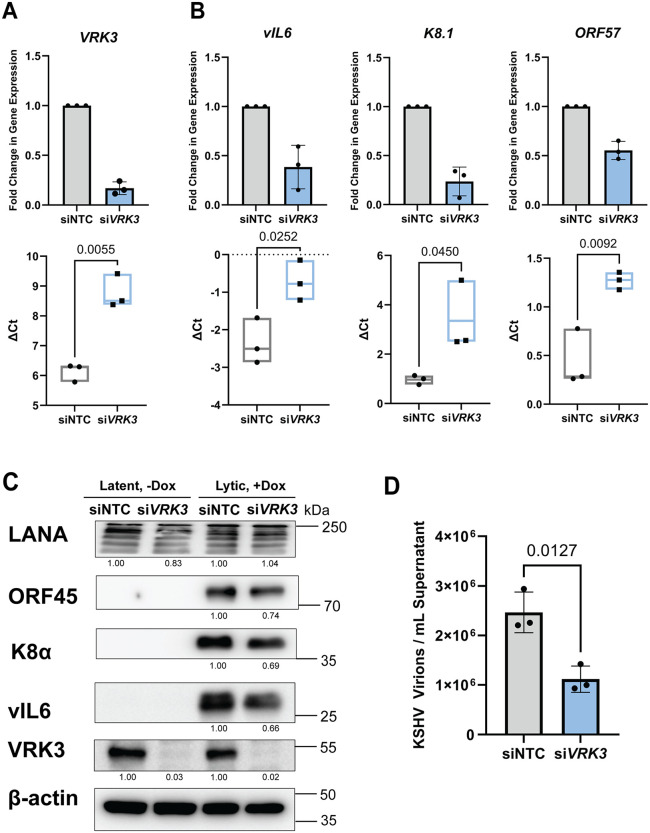
VRK3 promotes KSHV reactivation in PEL. The primary effusion lymphoma cell line TREx-BCBL1-RTA was electroporated with pooled siRNA targeted to VRK3 or a non-targeting control, then 48 hours post-electroporation, was reactivated with 1 µg/mL doxycycline for 48 hours. Cell pellets were collected for RNA extraction for RT-qPCR and cell lysates were collected for immunoblot (n = 3). **(A)** VRK3 depletion was confirmed by RT-qPCR (n = 3). Fold change in gene expression is shown and error bars indicate the standard error from the mean of three biological replicates. p-values were analyzed by a student’s t-test from ΔCt values. **(B)** KSHV viral transcripts *vIL6*, *K8.1*, and *ORF57* were quantified by RT-qPCR (n = 3). Fold change in gene expression is shown and error bars indicate the standard error from the mean of three biological replicates. p-values were analyzed by a student’s t-test from ΔCt values. **(C)** Latent samples were collected prior to reactivation and compared to samples collected 48 hours post-reactivation. KSHV viral proteins LANA, ORF45, K8a, and vIL6 expression were detected by immunoblot (n = 3). **(D)** At 48 hours post-reactivation, supernatant was collected to quantify extracellular virions by RT-qPCR relative to a standard curve of KSHV ORF39 plasmid (n = 3). p-values were analyzed by a student’s t-test and error bars indicate the standard error from the mean of three biological replicates.

We next investigated whether VRK3 was important for reactivation of KSHV from B cells. KSHV is the causative agent of primary effusion lymphoma (PEL), a B cell lymphoma. All PEL are infected with KSHV. We utilized the PEL cell line, TREx-BCBL1-RTA cells, which is a derivative of the patient-derived BCBL1 PEL cell line. Similar to the iSLK.219 cells, TREx-BCBL1-RTA cells are under the control of a tetracycline-inducible promoter for expression of RTA for lytic reactivation [31]. VRK3 expression was depleted in TREx-BCBL1-RTA cells via pooled siRNA electroporation, and 48 hours later, the cells were reactivated with doxycycline. Forty-eight hours following doxycycline treatment of the TREx-BCBL1-RTA cells, we measured KSHV reactivation markers. At 48 hours post-reactivation, we confirmed VRK3 knockdown (Fig 3A). Comparing cells transfected with VRK3 siRNA to cells transfected with control NTC siRNA, we observed a broad decrease in viral gene expression of the *vIL6*, *K8.1*, and *ORF57* mRNA transcripts in VRK3-depleted cells (Fig 3B). We also compared viral protein expression in latent and lytic cells that were transfected with either NTC siRNA or VRK3 siRNA. For both siRNA conditions, we observed only LANA was expressed during latency. However, during lytic reactivation, comparing cells transfected with VRK3 siRNA to cells transfected with NTC siRNA, we saw a reduction in viral lytic proteins ORF45, K8α, and vIL6 in VRK3-depleted cells (Fig 3C). Concomitant with these results, in VRK3 knockdown cells, the number of virions secreted into the supernatant was also decreased relative to the control (Fig 3D). Thus, VRK3 is also required for optimal reactivation of KSHV from PEL.

### VRK3 expression is increased and localizes to the nucleus during reactivation

**Fig 4:**
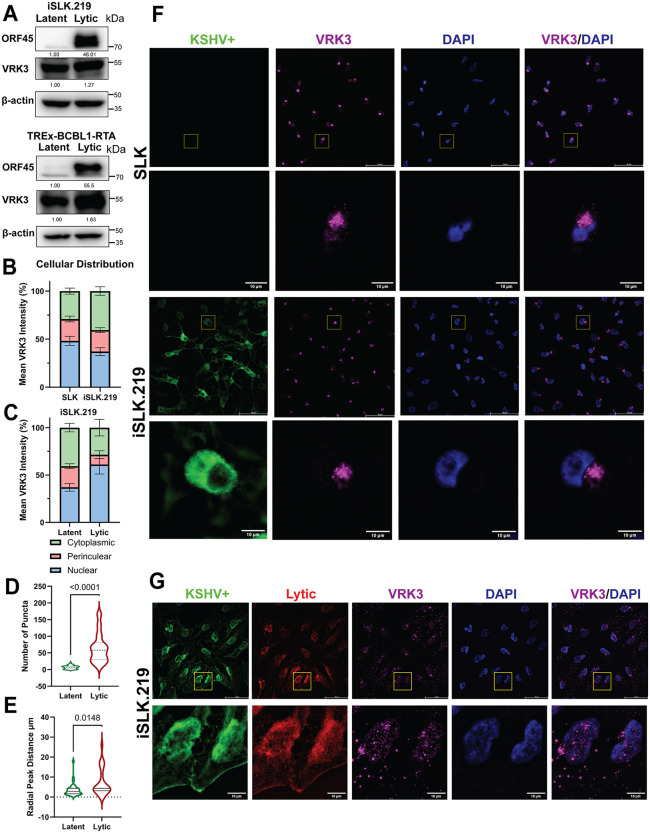
KSHV lytic reactivation upregulates VRK3 and re-localizes VRK3 to the nucleus. **(A)** iSLK.219 and TREx-BCBL1-RTA cells were reactivated with 1 µg/mL doxycycline (n = 3 for both cell types). Latent and lytic cell lysates were harvested and VRK3 expression was examined by immunoblot in iSLK.219 (upper panel) and TREx-BCBL1-RTA cells (lower panel). For Fig 4B-4G, VRK3 expression was examined in uninfected SLK, latent iSLK.219, and lytic iSLK.219 cells by immunofluorescence assay and subsequently analyzed. **(B)** In SLK and latent iSLK.219 cells, the distribution of VRK3 signal in the cytoplasm, perinuclear region, and nucleus in each cell was quantified for each cell population by percentage (n = 3). Error bars indicate the standard error of the mean of three biological replicates. **(C)** In latent and lytic iSLK.219 cells, the distribution of VRK3 signal in the cytoplasm, perinuclear region, and nucleus in each cell was quantified for each cell population by percentage (n = 3). Error bars indicate the standard error of the mean of three biological replicates. **(D)** In latent and lytic iSLK.219 cells, the number of VRK3 puncta per cell was quantified by Laplacian of Gaussian blob detection for each cell population (n = 3). The average for latent iSLK.219 cells was 7 puncta/cell and the average for lytic iSLK.219 cells was 58 puncta/cell. Three biological replicates were analyzed for VRK3 puncta counts per cell, and the p-value was determined by a student’s t-test. **(E)** For latent and lytic iSLK.219 cells, the radial peak distance of VRK3 signal was measured (n = 3). The VRK3 median radial peak distance for latent cells was 3.06 µm and for lytic cells was 4.75 µm. Radial peak distances within each cell from cell populations across three biological replicates were analyzed and the p-value was determined by student’s t-test. **(F)** Immunofluorescence assay of VRK3 expression in uninfected SLK cells compared to KSHV-infected latent iSLK.219 cells (n = 3). Images are representative of three biological replicates. **(G)** Immunofluorescence assay of VRK3 expression in iSLK.219 cells 48 hours post-reactivation (n = 3). Images are representative of three biological replicates.

We observed that VRK3 expression is upregulated during both primary KSHV infection in HUVEC cells and lytic reactivation in both iSLK.219 and TREx-BCBL1-RTA cells (Figs 1A and 4A).

Given prior reports that VRK3 localizes to both the nucleus and cytoplasm [5,24,25], we next examined whether KSHV infection alters VRK3 subcellular distribution. A previously published interactome analysis has shown that VRK3 depletion preferentially impacts metabolic pathways, and that VRK3 interacts with proteins associated with the endoplasmic reticulum (ER) and mitochondria, highlighting a potential role in coordinating cellular metabolism and organelle function [28].

Under basal conditions, we found that VRK3 predominantly localizes in a tightly clustered perinuclear pattern, at the perimeter of DAPI and Lamin B1 staining (Fig 4F and S2C Fig). Immunofluorescence assays showed that VRK3 is at the nuclear periphery and by organelle colocalization analysis, primarily colocalizes with the ER marker protein disulfide isomerase (PDI) by index of correlation (Fig 4F and S2E and S2G Fig). Both uninfected SLK cells and KSHV-infected latent iSLK.219 cells exhibited similar VRK3 localization profiles, where the average percent of VRK3 signal at the perinuclear region, bounded by a 2 µm fixed expansion from the nuclear edge defined by DAPI, was 22.8% for SLK cells and 22.3% for iSLK.219 cells (Fig 4B and 4F). Notably, upon induction of lytic reactivation in iSLK.219 cells, VRK3 distribution became more diffuse, accompanied by increased nuclear localization and punctate staining patterns (Fig 4C-4E, 4G). Comparing lytic to latent iSLK.219 cells, we observed that lytic cells have 8-fold more puncta, by Laplacian of Gaussian (LoG) blob detection (Fig 4D). Examining the radial distribution of VRK3 signal, the median peak distance for latent cells is 3.06 µm and lytic cells is 4.75 µm, indicating latent VRK3 clusters are concentrated closer to the nucleus, whereas lytic VRK3 is more diffused (Fig 4E).

This dynamic upregulation and relocalization of VRK3 during KSHV infection raises the possibility that VRK3 performs distinct, stage-specific functions during latency and lytic reactivation. Perinuclear and ER-associated VRK3 during latency may support metabolic homeostasis, whereas cytosolic distribution and nuclear accumulation during lytic reactivation could reflect a bigger role in regulating transcriptional programs or suppressing innate immune signaling that facilitate viral replication. During latency, VRK3 is most abundant in the perinuclear region but it is also present in the nucleus and is diffused throughout the cytoplasm. During lytic reactivation, VRK3 expression is increased in the nucleus, which may be a reflection of the KSHV-mediated increase in VRK3 expression. Together, these observations suggest that KSHV may actively modulate VRK3 expression and localization.

### VRK3 inhibits the antiviral type I interferon response

Loss of VRK3 led to an overall downregulation of KSHV infection and reactivation. Viral infections often trigger a type I interferon response that limits pathogenesis. cGAS-STING and RLR signaling are key innate immunity pathways that have been shown to restrict KSHV [32]. cyclic GMP-AMP Synthase (cGAS) is a DNA sensor that can detect and bind KSHV DNA [32,33], which leads to production of the second messenger molecule 2’3’-cyclic GMP-AMP (cGAMP) that activates adaptor protein Stimulator of Interferon Genes (STING). Retinoic acid-inducible gene I (RIG-I)-like receptors (RLRs), RIG-I and melanoma differentiation-associated protein 5 (MDA5), are both RNA sensors that can detect and bind KSHV-related RNA [34,35]. Both RNA sensors signal through the adaptor protein mitochondrial antiviral-signaling protein (MAVS). Adaptor proteins, STING and MAVS, trigger the signaling cascade that phosphorylates TANK-binding Kinase 1 (TBK1), which then phosphorylates transcription factor Interferon Regulatory Factor 3 (IRF3). IRF3 homodimerizes and translocates to the nucleus to drive the transcription of the antiviral response through type I interferons (S4A Fig).

To combat innate immunity, KSHV relies on viral and host factors to evade the antiviral innate immune response. KSHV tegument protein, ORF52 (KicGAS), inhibits cGAS by directly binding to both DNA and cGAS, preventing cGAS from sensing KSHV DNA [36]. In addition, cytoplasmic isoforms of KSHV LANA bind to cGAS to inhibit downstream cGAS-STING signaling, which allows LANA to promote reactivation from latency [37]. ORF33 antagonizes STING by recruiting the cellular phosphatase, PPM1G, to dephosphorylate STING, terminating subsequent signaling [38]. Another study showed that KSHV microRNAs bind STING mRNA transcript, to repress its translation, inhibiting downstream antiviral responses [39]. Additionally, KSHV inhibits RIG-I signaling by encoding deubiquitinase, ORF64, which targets RIG-I for proteasomal degradation [40,41]. Furthermore, KSHV uses cellular factor, adenosine demaniase acting on RNA 1 (ADAR1), to dampen RLR-signaling for optimal lytic reactivation [42,43]. KSHV encodes viral Interferon Regulatory Factors (vIRFs) that are homologs of host interferon regulatory factors (IRFs). vIRF1 inhibits the type I interferon response by binding IRF3, which prevents both IRF3 activation by phosphorylation and its subsequent translocation to the nucleus [44]. It has also been reported that KSHV uses cellular caspase-dependent mechanism to attenuate type I interferon responses, where several caspases including caspase-3 and -8 are upregulated during KSHV lytic reactivation to suppress IFNβ induction [45]. Thus, we examined the impact of VRK3 on the type I interferon response.

**Fig 5:**
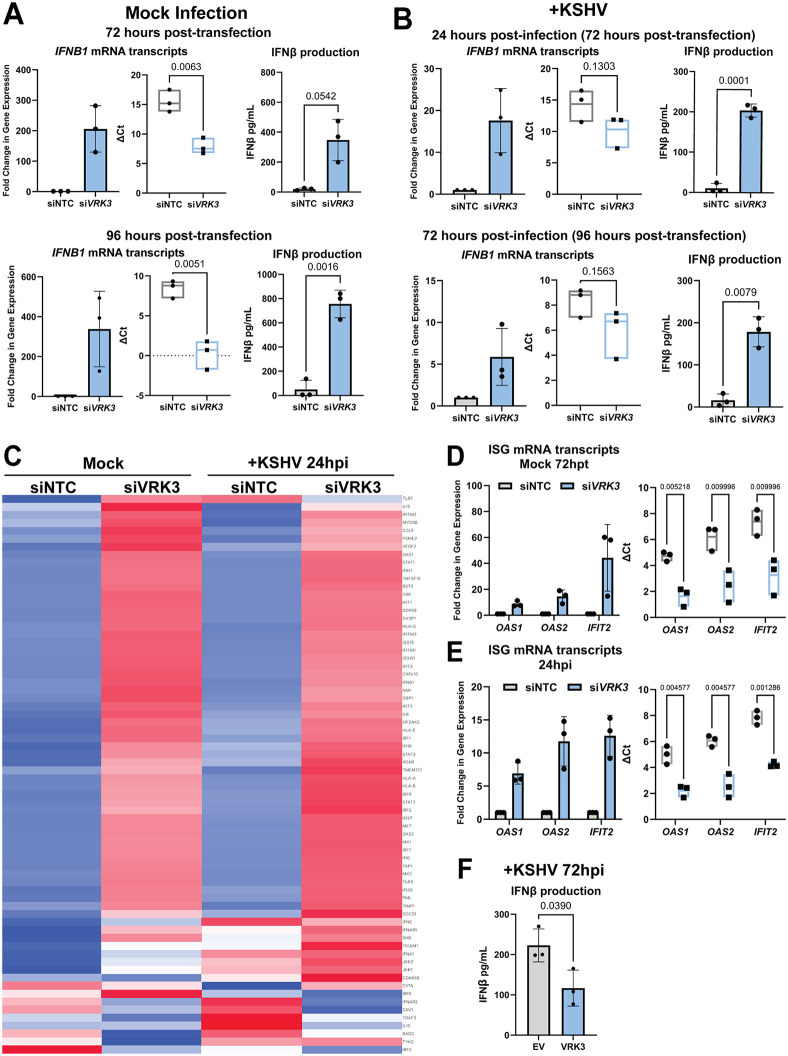
VRK3 restricts the antiviral type I interferon response. For Fig 5A-5E, HUVEC cells were depleted with non-targeting control or VRK3-targeted pooled siRNA. Twenty-four hours post-transfection, cells were either mock or KSHV-infected and harvested at 72- or 96-hours post-siRNA transfection (n = 3). **(A)** Mock-infected HUVEC cells were analyzed for *IFNB1* mRNA transcripts by RT-qPCR and IFNβ production by ELISA at 72- and 96-hours post-siRNA transfection (n = 3). For *IFNB1* transcripts, fold change in gene expression is shown and error bars indicate the standard error from the mean of three biological replicates. p-values were analyzed by a student’s t-test from ΔCt values. For IFNβ ELISAs, p-values were analyzed by student’s t-test and error bars indicate the standard error from the mean of three biological replicates. **(B)** KSHV-infected HUVEC cells were analyzed for *IFNB1* mRNA transcripts by RT-qPCR and IFNβ production by ELISA at equivalent timepoints for hours post-transfection as (A) at 24- and 72-hours post-infection (n = 3). For *IFNB1* transcripts, fold change in gene expression is shown and error bars indicate the standard error from the mean of three biological replicates. p-values were analyzed by a student’s t-test from ΔCt values. For IFNβ ELISAs, p-values were analyzed by student’s t-test and error bars indicate the standard error from the mean of three biological replicates. **(C)** RT^2^ Profiler Array for Human Type I Interferon Response (Qiagen, Cat# PAHS-016Z) was assayed according to manufacturer’s instructions for mock- and KSHV-infected HUVEC cells transfected with non-targeting control siRNA or VRK3 at 72 hours post-transfection (n = 2). **(D)** Mock-infected HUVEC cells were analyzed for a panel of interferon-stimulated genes, *OAS1, OAS2*, and *IFIT2* mRNA transcripts by RT-qPCR at 72 hours post-transfection (hpt) (n = 3). Fold change in gene expression is shown and error bars indicate the standard error from the mean of three biological replicates. p-values were analyzed by multiple t-test from ΔCt values. **(E)** KSHV-infected HUVEC cells were analyzed for a panel of ISGs, *OAS1, OAS2*, and *IFIT2*, mRNA transcripts by RT-qPCR at 72 hours post-transfection/24 hours post-infection (hpi) (n = 3). Fold change in gene expression is shown and error bars indicate the standard error from the mean of three biological replicates. p-values were analyzed by multiple t-test from ΔCt values. **(F)** HUVEC cells were transfected with either an empty vector control or VRK3 expression plasmid for 48 hours, then infected with rKSHV.219 for 72 hours. At 72 hours post-infection, supernatants were collected and IFNβ production was analyzed by ELISA (n = 3). p-values were analyzed by student’s t-test and error bars indicate the standard error from the mean of three biological replicates.

VRK3 or control NTC pooled siRNA was transfected into HUVEC cells for 72 and 96 hours. We found that *IFNB1* mRNA transcripts and IFNβ production were increased in the VRK3 siRNA transfected cells compared to the NTC siRNA transfected cells (Fig 5A). Similarly, when VRK3 was depleted in HUVEC cells and then subsequently infected with KSHV for either 24 or 72 hours, we also saw an upregulation of *IFNB1* mRNA transcripts and IFNβ production (Fig 5B). Next, we profiled the impact of VRK3 depletion on interferon stimulated genes (ISGs) using a RT-qPCR profiler array for type I interferon genes. We observed a broad upregulation of type I interferon genes in VRK3 siRNA transfected cells relative to our NTC siRNA transfected cells in both mock and KSHV-infected conditions (Fig 5C). We examined a panel of ISGs including *OAS1, OAS2,* and *IFIT2*, and found these ISGs were all increased when VRK3 was depleted either with or without infection (Fig 5D and 5E). Conversely, we overexpressed VRK3 by transfecting either an empty vector (EV) or VRK3 expression plasmid into HUVEC cells for forty-eight hours, then infected with rKSHV.219. Seventy-two hours post-infection, we measured IFNβ levels by ELISA and found that VRK3 overexpression relative to the EV control significantly reduced IFNβ production (Fig 5F). Suppressing the type I interferon response is a novel role for VRK3. VRK3 is distinct from VRK1 in this regard [18], where we found that VRK1 knockdown does not impact *IFNB1* mRNA transcripts relative to the control in mock- or KSHV- infected cells (S3A and S3B Fig). Furthermore, pooled siRNA results were consistent with individual sequence-targeted siRNAs, where individual VRK3 siRNA transfected cells (compared to NTC siRNA transfected cells) also led to a significant increase in interferon production by IFNβ ELISA in both mock and KSHV infection conditions (S3C and S3D Fig).

We also examined whether the contribution of VRK3 to KSHV infection was dependent upon the function of VRK3 suppressing type I interferons. In HUVEC cells, we depleted expression of VRK3 by pooled siRNA and treated with either isotype control or IFNβ neutralizing antibodies for 24 hours, then infected with rKSHV.219 for 72 hours. Seventy-two hours post-infection, we validated the neutralizing activity of the IFNβ antibody treatment and analyzed the impact on KSHV infection by quantitating the number of GFP-positive cells. We confirmed VRK3 knockdown by immunoblot and probed STAT1, a downstream IFNβ-target that is phosphorylated in response to IFN activation. We observed that phospho-STAT1 (Tyr701) was reduced, confirming effective IFNβ neutralizing antibody treatment in both the NTC and VRK3 siRNA conditions (S5C Fig). In the NTC siRNA condition, treatment with isotype control or IFNβ neutralizing antibodies made no significant difference to KSHV infection as measured by GFP-positive cells (S5A Fig). Comparing cells transfected with VRK3 siRNA to cells transfected with NTC siRNA, treatment with the isotype control in VRK3-depleted cells led to a significant decrease in KSHV infection (S5A Fig), similar to our previous studies (Fig 1B-1D). In VRK3 siRNA transfected cells, the IFNβ neutralizing antibody treatment (compared to treatment with the isotype control) restored KSHV infection levels by GFP-positive cells, with infection levels similar to NTC siRNA transfected cells (S5A Fig). This is further supported by siRNA studies where IFNB1 was depleted in combination with VRK3 in HUVEC cells to a similar effect observed with IFNβ neutralizing antibody treatment. STAT1 phosphorylation was decreased in both the IFNB1 single and combination knockdowns (S5D Fig). The combination knockdown of VRK3 and IFNB1 restored KSHV infection to levels similar to NTC knockdown, as measured by GFP-positive cells (S5B Fig). Given that KSHV infection is restored by blocking interferon, our data demonstrates that VRK3 promotes KSHV infection by suppressing the antiviral type I interferon response.

### VRK3 negatively regulates the TBK1-IRF3 signaling axis

**Fig 6:**
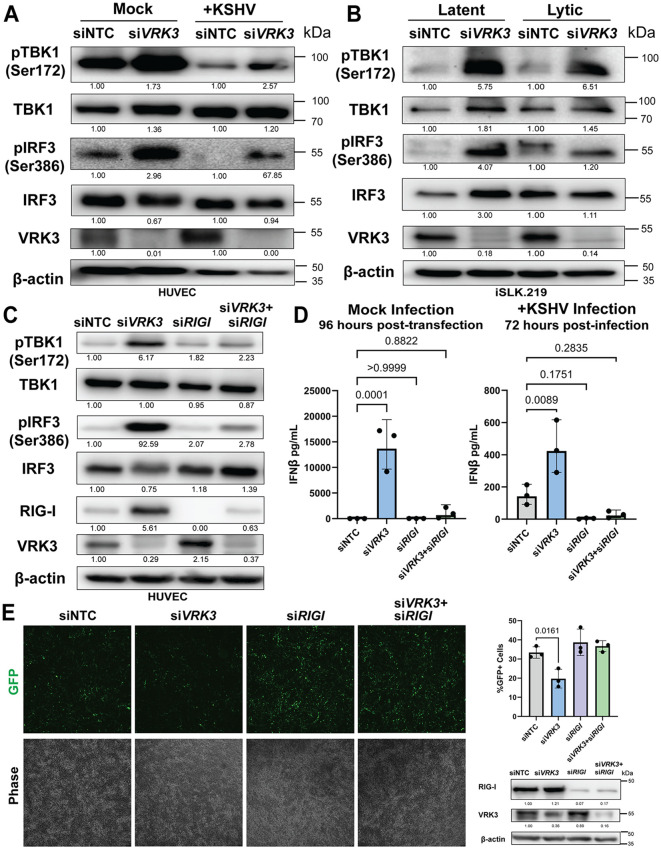
VRK3 negatively regulates the TBK1-IRF3 signaling axis. **(A)** HUVEC cells were depleted of VRK3 by pooled siRNA for 24 hours then were either mock or KSHV infected for 72 hours. Activation of TBK1 (Ser172) and IRF3 (Ser386) were probed for by immunoblot. The blot was subsequently stripped and then re-probed for total protein expression of TBK1 and IRF3, after which the blot was stripped and re-probed to confirm VRK3 knockdown and β-actin was used as the loading control (n = 3). **(B)** Latent iSLK.219 cells were depleted of VRK3 by pooled siRNA for 48 hours and phosphorylation of TBK1 and IRF3 was measured. Additionally, VRK3-depleted cells were reactivated for 72 hours and probed for phosphorylated TBK1 and IRF3. Blots were stripped and re-probed for total protein expression of TBK1 and IRF3. Blots were then stripped and re-probed for VRK3 to confirm knockdown. β-actin was used as a loading control (n = 3). **(C)** HUVEC cells were transfected with either non-targeting control (NTC), VRK3, RIG-I, or a combination of VRK3 and RIG-I pooled siRNA for 48 hours. Phosphorylation and activation of TBK1 (Ser172) and IRF3 (Ser386) were determined by immunoblotting. The blot was subsequently stripped and then re-probed for total protein expression of TBK1 and IRF3, after which the blot was stripped and re-probed to confirm RIG-I and VRK3 knockdown. β-actin was used as the loading control (n = 3). For Fig 6D-6E, HUVEC cells were transfected with either non-targeting control, VRK3, RIG-I, or VRK3 and RIG-I combined pooled siRNA. Twenty-four hours post-transfection, cells were either mock or KSHV-infected and cells were harvested 72 hours post-infection (96-hours post-siRNA transfection) (n = 3). **(D)** Mock- and KSHV- infected HUVEC cells were analyzed for IFNβ production by ELISA (n = 3). p-values were determined using a one-way ANOVA test and error bars indicate the standard error from the mean of three biological replicates. **(E)** KSHV infection was analyzed by observing GFP^+^ cells using fluorescence microscopy and the GFP^+^ cells were also quantified by flow cytometry at 72 hours post-infection (n = 3). p-values were determined by a one-way ANOVA test and error bars indicate the standard error from the mean of three biological replicates. Cell lysates were prepared and analyzed by immunoblot to validate RIG-I and VRK3 knockdown (n = 3).

We next sought to define how VRK3 influences signaling events that regulate type I interferon production. Many innate immune pathways depend on the activation of the TBK1-IRF3 axis [46–48]. TBK1 is a serine/threonine kinase activated by phosphorylation that is a critical node in signaling between adaptor proteins and transcription factors like IRF3 to drive interferon production. We examined activation of both TBK1 and IRF3 as measured by their phosphorylation status and found that when VRK3 was depleted in the HUVEC cells in the context of either mock or KSHV infection, phosphorylation of TBK1 (Ser172) and IRF3 (Ser386) was increased relative to NTC siRNA transfected cells (Fig 6A). During KSHV reactivation in the iSLK.219 cells, we also found that VRK3 knockdown compared to NTC siRNA transfected cells led to an increase in pTBK1 and pIRF3 (Fig 6B). Thus, it appears that VRK3 acts as a suppressor of TBK1-IRF3 signaling in mock infected cells, primary infection, and in reactivation.

We investigated how VRK3 regulates TBK1-IRF3 signaling using an IFNβ-luciferase assay. HEK293T cells express cGAS but not STING. Hence, in the absence of STING, these cells do not activate the IFN pathway or IFNβ promoter [49,50]. We performed an IFNβ-luciferase assay with HEK293T cells to measure IFNβ promoter activation and transfected in an empty vector or VRK3 expression plasmid along with components of the cGAS-STING pathway (S4A Fig) either individually or together. When we transfected expression plasmids for cGAS, STING, TBK1, or IRF3 individually, with an empty vector (EV) control or VRK3, there was no statistically significant difference in the activation of the IFNβ promoter. Co-expression of VRK3 with cGAS+STING led to a slight reduction in IFNβ-luciferase activity relative to the empty vector control (S4B Fig). We next examined the impact of VRK3 on the production of the second messenger molecule, cGAMP, as a readout for cGAS-STING pathway activation in physiologically relevant cell lines. In basal conditions, cGAMP levels in the HUVEC cells transfected with VRK3 or NTC control siRNA were below the assay limit of detection (9.6 pg/mL). We also examined iSLK.219 cells, and we found that cells transfected with VRK3 siRNA relative to cells transfected with NTC siRNA had no significant effect on cGAMP production, indicating VRK3 does not mediate a direct effect on cGAS or cGAMP (S4C Fig). However, when we examined the effect of VRK3 depletion in HUVEC cells on phosphorylation of STING at serine 366 by immunoblot, we found that cells transfected with VRK3 increases both phospho-STING and total STING protein expression relative to cells transfected with NTC siRNA (S4D Fig).

We next examined whether STING was the target of VRK3. We did a combination depletion of VRK3 and STING using pooled siRNA and compared this condition to non-targeting control siRNA transfected cells as well as siRNA depletions of either VRK3 or STING in HUVEC cells after 48 hours. Upon examination of TBK1 and IRF3 activation, we found that combination knockdown of STING and VRK3 did not restore phosphorylated levels of TBK1 and IRF3 to baseline relative to the NTC control or STING knockdown alone (S4E Fig). Comparing the combination knockdown of VRK3 and STING relative to single knockdown of VRK3, the phosphorylation of TBK1 and phosphorylation of IRF3 are similar (S4E Fig), suggesting that VRK3 is not directly regulating cGAS or STING.

### VRK3 promotes KSHV infection through RIG-I

We hypothesized that the elevated phosphorylated STING we observed upon VRK3 knockdown may be due to crosstalk between RNA and DNA sensing pathways. STING has been reported to transmit RIG-I-MAVS signals during viral infection [51]. Additionally, RIG-I-mediated STING upregulation restricts infection of another DNA virus, HSV-1 [52]. We examined the impact of VRK3 on RIG-I signaling. In HUVEC cells we depleted expression of VRK3 and RIG-I together using pooled siRNA and compared this condition to a non-targeting control as well as to single pooled siRNA depletions of either VRK3 or RIG-I. Under basal conditions, the combination knockdown of VRK3 and RIG-I ablates the activation of TBK1 and IRF3 that we observe from single knockdown of VRK3 (Fig 6C), suggesting that VRK3 mediates suppression of TBK1-IRF3 signaling in a RIG-I-dependent manner.

Based on this result, we investigated whether VRK3 suppression of RIG-I-interferon responses contributes to KSHV infection. We transfected HUVEC cells with NTC, VRK3, RIG-I, or VRK3 and RIG-I combined pooled siRNA for 24 hours, then subsequently infected with rKSHV.219 for 72 hours. Seventy-two hours post-infection, we validated knockdowns (Fig 6E) and examined the impact on interferon responses and KSHV infection. We measured IFNβ production by ELISA in both mock and KSHV-infected cells that had been depleted for VRK3 and RIG-I (Fig 6D). Similar to our earlier observations, VRK3 knockdown alone increased interferon production in mock and infected cells, while RIG-I knockdown displayed interferon production similar to the control knockdown cells. Comparing interferon production for the combination knockdown of VRK3 and RIG-I relative to the VRK3 single knockdown cells, IFNβ production was ablated in the combination VRK3 and RIG-I siRNA transfected cells. The VRK3 and RIG-I combination knockdown led to similar levels of IFNβ as both the NTC and RIG-I single knockdown cells (Fig 6D). These data support that VRK3 inhibition of type I interferon is through negative regulation of RIG-I. We next analyzed KSHV infection by measuring GFP-positive cells by fluorescence microscopy and flow cytometry. We found that cells transfected with a combination of VRK3 and RIG-I siRNA restored KSHV infection to similar levels as we observed with cells singly transfected with NTC siRNA (Fig 6E). Hence, our data suggests that VRK3 promotes KSHV infection by suppressing RIG-I-mediated type I interferon responses.

## Discussion

We show that VRK3 expression is consistently upregulated during de novo KSHV infection in endothelial cells and during lytic reactivation in epithelial and B cells. Depletion experiments demonstrate that VRK3 is required for efficient de novo KSHV infection of endothelial cells and for KSHV viral gene expression, genome replication, and production of infectious virions during reactivation in epithelial and PEL cells. Overexpression experiments demonstrate that VRK3 promotes KSHV primary infection and lytic reactivation. Notably, despite their homology, VRK1 and VRK3 exhibit distinct roles during infection, as VRK1 depletion did not impair KSHV infection. These findings underscore functional divergence within the VRK family and highlight VRK3 as a unique regulator of antiviral immunity.

Mechanistically, our data indicate that VRK3 likely suppresses the type I interferon response by inhibiting the TBK1-IRF3 signaling axis. Loss of VRK3 resulted in increased phosphorylation of TBK1 and IRF3, elevated *IFNB1* transcription, enhanced IFNβ production, and broad induction of interferon-stimulated genes, both in the presence and absence of infection. These effects were also observed during KSHV primary infection and lytic reactivation, suggesting that VRK3 functions as a constitutive brake on innate immune signaling rather than acting exclusively in response to viral stimuli. Furthermore, VRK3 overexpression decreased IFNβ production during KSHV infection. In the context of KSHV infection, VRK3-mediated inhibition of the type I interferon response is critical for the contribution of VRK3 to KSHV infection. In the context of VRK3 depletion, blocking type I interferons by neutralizing antibody or siRNA treatment restores KSHV infection to levels similar to the control cells. Importantly, we found that VRK3 mediates this in a RIG-I-dependent manner. Combination knockdown of VRK3 and RIG-I ablates activation of TBK1-IRF3 signaling observed in VRK3 single knockdown and restores IFNβ to levels similar to control cells.

KSHV encodes multiple viral interferon antagonists, including viral IRFs and microRNAs that target components of the innate immune machinery. Our findings suggest that KSHV additionally exploits a host-encoded interferon suppressor, VRK3, to reinforce immune evasion. The observation that VRK3 depletion enhances interferon signaling even in mock-infected cells further suggests that VRK3 may act as a general regulator of innate immunity, which KSHV co-opts to create a more KSHV-permissive intracellular environment.

**Fig 7:**
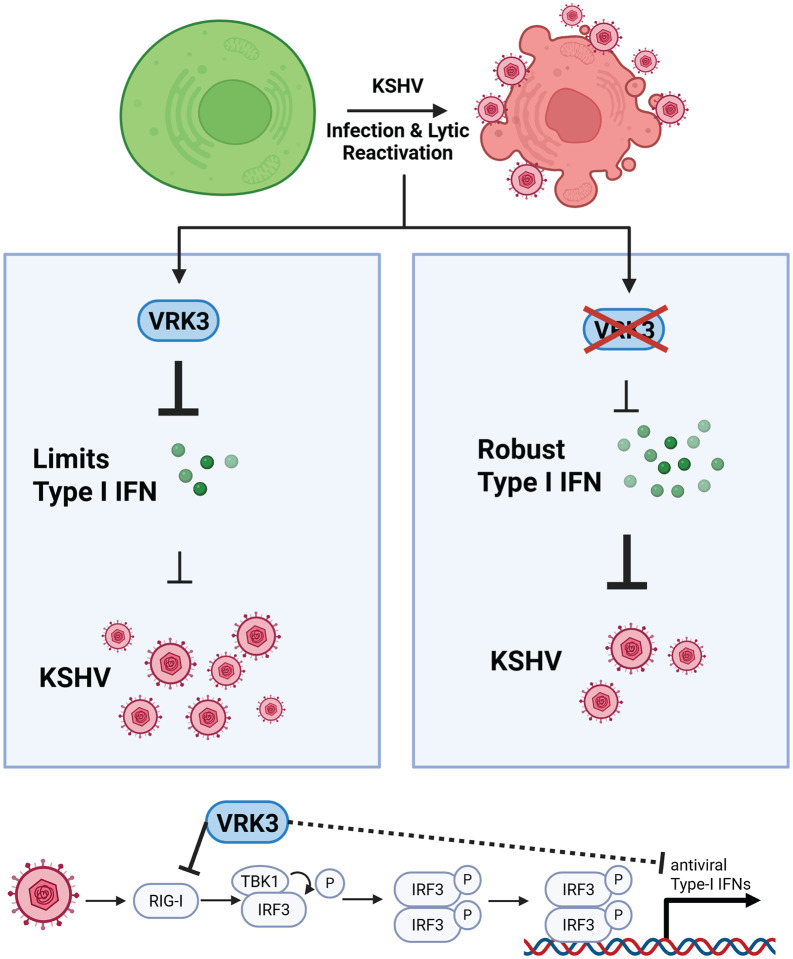
Proposed Model of VRK3 promoting KSHV. VRK3 contributes to KSHV infection and reactivation by suppressing the type I interferon response. VRK3 inhibits TBK1-IRF3 signaling in a RIG-Idependent manner.

The dynamic regulation of VRK3 expression and subcellular localization during infection further supports its functional importance. Under basal and latent conditions, VRK3 localized predominantly to perinuclear and ER-associated compartments, positioning VRK3 near key innate immune signaling hubs. Upon lytic reactivation, VRK3 became more diffuse and showed increased nuclear accumulation, suggesting that KSHV may spatially reprogram VRK3 to meet the changing demands of viral replication. In summary, this work identifies VRK3 as a novel host factor that promotes KSHV infection and reactivation by suppressing the type I interferon response through the TBK1-IRF3 axis (Fig 7). These findings expand our understanding of how KSHV co-opts host signaling pathways to evade innate immunity and highlight VRK3 as a potential target for therapeutic intervention in KSHV-associated diseases.

## Materials and methods

### Cell culture

hTERT-immortalized HUVEC cells were maintained in Endothelial Cell Growth Medium (PromoCell) containing the supplement kit (except ascorbic acid and heparin sulfate), 10% FBS (VWR), 1% penicillin-streptomycin (Thermo Fisher), and 1% L-glutamine (Thermo Fisher). HEK293, HEK293T, and SLK cells were maintained in DMEM (Thermo Fisher) containing 10% FBS, 1% penicillin-streptomycin, and 1% L-glutamine. iSLK.219 cells were maintained in DMEM containing 10% Tet-free FBS (TaKaRa), 1% penicillin-streptomycin, 1% L-glutamine, 10 μg/mL puromycin (Corning), 250 μg/mL Geneticin (Thermo Fisher), and 400 μg/mL hygromycin B (Corning). TREx-BCBL1-RTA cells (kindly gifted by Dr. Jae Jung) were maintained in RPMI 1640 (Corning) containing 10% Tet-free FBS, 1% penicillin-streptomycin, 1% L-glutamine, 1% sodium bicarbonate (Thermo Fisher), 0.05 mM β-mercaptoethanol (Thermo Fisher), and 200 μg/mL hygromycin. Cells were cultured in 37 °C and 5% CO2 incubators.

### Virus

KSHV (rKSHV.219) was isolated from lytically reactivated iSLK.219 cells. iSLK.219 cells were expanded and then stimulated for virus production in DMEM with 3 μg/mL doxycycline (Thermo Fisher) and 1 mM sodium butyrate (Sigma-Aldrich). Sixty hours after lytic induction, supernatants were harvested, centrifuged, and then filtered. Supernatant was layered over a 20% sucrose cushion. The virus was pelleted by ultracentrifugation in a SW32Ti rotor (Beckman Coulter) at 24,000 rpm for 2.5 hours at 4 °C. The pellets were resuspended in endotoxin-free PBS (Thermo Fisher).

### siRNA transfection and reactivation

SMARTpool siRNA (Horizon Discovery) targeting genes of interest (*BANF1*: L-011536, *IFNB1*: L-019656, *STING* (*TMEM173*): L-024333, *RIG-I* (*DDX58*): L-012511, *VRK1*: L-004683, *VRK3*: L-005397) or control (NTC: D-001810) were resuspended at 100 µM as stock solutions. Individual siRNA (Horizon Discovery) targeting genes of interest (*VRK3*: J-005397–06, J-005397–07, and J-005397–08) or control (NTC: D-001810–01) were resuspended at 100 µM as stock solutions. All siRNA sequences can be found in S1 Table.

siRNA transfections for HUVEC and iSLK.219 cells were performed with 20 nM of pooled siRNA in Lipofectamine RNAiMax (Thermo Fisher) in OptiMEM (Thermo Fisher) according to the manufacturer’s protocol. HUVEC cells were forward transfected where cells were seeded and then transfected 24 hours later. iSLK.219 cells were reverse transfected, where siRNA and transfection solution were mixed and added to the wells, after which 2 x 10^5^ cells/well were seeded in the wells. Forty-eight hours post-transfection, iSLK.219 cells were reactivated with 50 ng/mL doxycycline in culture media. TREx-BCBL1-RTA were transfected with 100 pmol of pooled siRNA per 1 x 10^6^ cells using the Neon NxT Electroporation system (Invitrogen) according to the manufacturer’s BC-1 protocol and published literature, and subsequently plated in culture media [53]. Forty-eight hours post-nucleofection, TREx-BCBL1-RTA were reactivated with 1 µg/mL doxycycline in culture media.

### Plasmid transfection and reactivation

For HUVEC and iSLK.219 cells, the experimental conditions were the same. 1.5 x 10^5^ cells/well were plated in a 6-well plate 24 hours prior to transfection. Cells were treated with a transfection mixture containing 2 µg of either pCMV-VRK3–3XFLAG or pCMV empty vector control per well and 6 µg of X-tremeGENE 9 (Sigma-Aldrich) in Opti-MEM (Thermo Fisher). For primary infection experiments, after 48 hours post-transfection, HUVEC cells were infected with rKSHV.219. For reactivation experiments, iSLK.219 cells were treated with 50 ng/mL doxycycline. Samples were harvested at select timepoints for downstream analysis.

### Primary infection of HUVECs

HUVEC cells were plated 3.5 x 10^5^ cells/well in a 6-well plate in culture media. Twenty-four hours later, media was replaced with 100 μL of concentrated cell-free KSHV or PBS for mock-infected wells in a total well volume of 2.5 mL serum-free Endothelial Cell Growth Medium (PromoCell), supplemented with 10 μg/mL polybrene (Sigma-Aldrich). The cells were then spun at 2500 rpm at 30 °C for 90 minutes. Following spinfection, the media was replaced with Endothelial Cell Growth Medium with 10% FBS and 1% L-glutamine.

For primary infection experiments with pooled or individual siRNA-treated cells, 2.5 x 10^5^ HUVEC cells/well were plated in a 6-well in culture media. Twenty-four hours later, the HUVEC cells were forward transfected with a total concentration of 20 nM pooled siRNA (Horizon Discovery) using Lipofectamine RNAiMax in Opti-MEM (Thermo Fisher) according to the manufacturer’s protocol. After 24 hours, media was replaced with 100 μL concentrated cell-free KSHV or PBS for mock-infected wells in a total well volume of 2.5 mL serum-free Endothelial Cell Growth Medium (PromoCell), supplemented with 10 μg/mL polybrene (Sigma-Aldrich). The cells were then spun at 2500 rpm at 30 °C for 90 minutes. Following spinfection, the media was replaced with Endothelial Cell Growth Medium with 10% FBS and 1% L-glutamine.

For primary infection experiments with IFNβ neutralizing antibody treatment, 2.5 x 10^5^ HUVEC cells/well were plated in a 6-well plate in culture media. Twenty-four hours later, the HUVEC cells were forward transfected with a total concentration of 20 nM pooled siRNA (Horizon Discovery) using Lipofectamine RNAiMax (Thermo Fisher) in Opti-MEM (Thermo Fisher) according to the manufacturer’s protocol and treated with either 10 µg/mL of an isotype control antibody (MOPC-21) (Invitrogen, Cat# MA1–10407) or IFNβ monoclonal antibody (MMHB-3) (Invitrogen, Cat# 214001). After 24 hours, media was replaced with 100 μL concentrated cell-free KSHV or PBS for mock-infected wells in a total well volume of 2.5 mL serum-free Endothelial Cell Growth Medium (PromoCell), supplemented with 10 μg/mL polybrene (Sigma-Aldrich). The cells were then spun at 2500 rpm at 30 °C for 90 minutes. Following spinfection, the media was replaced with Endothelial Cell Growth Medium with 10% FBS and 1% L-glutamine and replenished with either 10 µg/mL isotype control or IFNβ monoclonal antibody.

### DNase-resistant viral genome assay

Supernatants were harvested from treated cells and then were passed through a 0.45 μm filter (Millipore Sigma). Supernatants were then treated with TURBO DNase in 1X TURBO DNase Buffer (Thermo Fisher) at 37 °C for 1 h. DNase treatment was inactivated with 10 mM EDTA (Corning) at 70 °C for 15 min. DNA was extracted using a DNEasy Blood and Tissue Kit (Qiagen) according to the manufacturer’s protocol. Real-time qPCR amplification of KSHV ORF39 in SensiFast Lo-Rox SYBR (Bioline) with a final primer concentration of 500 nM was performed on a QuantStudio 6 Flex Real-Time PCR System to quantify genome copy number. Standard curves were created using dilutions of pCDNA4/TO-ORF39 − 2XStrep (kindly gifted by Dr. Britt Glaunsinger). Primers used for qPCR are found in S2 Table. Genome copy numbers were compared statistically using student’s t-tests to obtain p-values.

### Viral supernatant transfer infectivity assay

HEK293 cells were plated at 5 x 10^5^ cells/well in 6-well plates with DMEM (Thermo Fisher) containing 10% FBS (VWR) and 1% L-glutamine. Twenty-four hours later, supernatants were collected from reactivated cells and centrifuged at 1500 rpm to pellet cells and debris. Equal volumes of supernatant were transferred to the naive HEK293 cells. The final volume of each well was brought up to 2.5 mL with serum-free DMEM and supplemented with 10 μg/mL polybrene (Sigma-Aldrich). The supernatants were spun with the naïve cells at 2500 rpm for 90 minutes at 30°C. Wells were supplemented with DMEM containing 10% FBS (VWR) and 1% L-glutamine and incubated overnight at 37 °C and 5% CO2 before the media was changed to 2 mL DMEM containing 10% FBS (TaKaRa) and 1% L-glutamine. Forty-eight hours post-supernatant transfer, GFP^+^ cells were quantified by fluorescent microscopy on a Leica Dmi8-inverted microscope. Subsequently, the cells were harvested and GFP^+^ cells were quantified by flow cytometry using a MACS-Quant VYB. GFP^+^ cell populations were analyzed by FlowJo. Relative sizes of cell populations were compared statistically using a student’s t-tests.

### Fluorescence microscopy and GFP/RFP quantification

All microscopy images were taken using a Leica Dmi8-inverted microscope. Images shown are representative of three biological replicates. Percentage of RFP^+^ cells were quantified using a MACS-Quant VYB. GFP/RFP^+^ cell populations were gated based on negative control samples using FlowJo.

### Immunofluorescence assays

SLK, iSLK.219 and latently infected HUVEC cells were seeded at 5 x 10^4^ cells/coverslip on glass coverslips. Prior to fixation, iSLK.219 cells were reactivated with doxycycline for 48 hours. On the day of harvest, cells were washed three times with 1X PBS and fixed with 4% paraformaldehyde for 15 minutes at room temperature with gentle shaking. Cells were then washed three times with 1X PBS. Cells were permeabilized and blocked in buffer (5% normal goat serum, 0.5% Tween 20, 5% glycine, in 1X PBS) with 0.3% Triton X-100 for 30 minutes at room temperature with gentle shaking. Primary antibodies were diluted in blocking buffer at 1:100 and incubated on the coverslips for 2 hours at room temperature with gentle shaking. Coverslips were washed three times with 1X TBST. Secondary antibody was diluted at 1:500 in blocking buffer and incubated onto coverslips for 1 hour at room with gentle shaking. Cells were washed with 1X TBST then rinsed with water. Coverslips were mounted on slides with ProLong Glass antifade with NucBlue. Coverslips were allowed to be cured on glass slides overnight, protected from light exposure. Cells were imaged with a 63x oil immersion lens on the Leica Dmi8 inverted microscope and processed with the Leica LAS X and ImageJ software. Immunofluorescence assay analysis of cell populations from three biological replicates of cellular compartment distribution, puncta counts, and radial distribution was conducted with CellProfiler and Biomni. The perinuclear ring was bounded by a 2 µm fixed expansion from the nuclear edge defined from DAPI staining. Puncta detection was from Laplacian of Gaussian blob detection with 0.5-3 µm radius. Dispersion was measured by radial peak distance where mean peak intensity was a function of distance from nuclear edge, binned by 1 µm steps to cell boundary as defined by GFP signal. For organelle colocalization, index of correlation coefficients were analyzed in ImageJ (Colocalization-ColorMap ImageJ plugin).

Antibodies used: VRK3 (Santa Cruz Biotechnology), Organelle Localization Sampler Kit (Cell Signaling Technology), Alexa Fluor 647 goat anti-mouse (Invitrogen).

### Immunoblotting

Cell pellets were lysed in RIPA buffer. Cell lysates were sonicated and centrifuged at 15,000 rpm for 10 min at 4°C to pellet cell debris. Lysate protein concentration was quantitated by BCA assay (Thermo Fisher). Immunoblot samples with equal protein concentrations were prepared in 5X urea and denatured for 5 minutes at 95°C. Samples were separated by SDS-PAGE and transferred to PVDF membrane (Thermo Fisher). Membranes used to probe phospho-targets were blocked and probed in 5% bovine serum albumin in TBST. For all other targets, the membrane was blocked with 5% nonfat milk in TBST. Membranes were incubated on a rocker with primary antibodies overnight at 4 °C. Antibodies used:

Human: β-actin-HRP (C4) mouse monoclonal (Santa Cruz Biotechnology, Cat.# sc-47778), vinculin-HRP (E1E9V) rabbit monoclonal (Cell Signaling Technology, Cat.#18799), RIG-I (D14G6) rabbit monoclonal (Cell Signaling Technology, Cat.# 3743), phospho-STAT1 Tyr701 (58D6) rabbit monoclonal (Cell Signaling Technology, Cat.# 9167), STAT1 (D1K9Y) rabbit monoclonal (Cell Signaling Technology, Cat.#14994), phospho-STING Ser366 rabbit polyclonal (Invitrogen, Cat.# PA5–105674), STING (D2P2F) rabbit monoclonal (Cell Signaling Technology, Cat.# 13647)phospho-TBK1 Ser172 (D52C2) rabbit monoclonal (Cell Signaling Technology, Cat.# 5483), TBK1 (D1B4) rabbit monoclonal (Cell Signaling Technology, Cat.# 3504), phospho-IRF3 Ser386 (EPR2346) rabbit monoclonal (Abcam, Cat.# Ab76493, rabbit), IRF3 (D83B9) rabbit monoclonal (Cell Signaling Technology, Cat.# 4302), VRK1 (1F6) mouse monoclonal (Cell Signaling Technology, Cat.# 3307), VRK2 rabbit polyclonal (Proteintech, Cat.# 12946–1-AP), VRK3 rabbit polyclonal (Proteintech, Cat.# 15608–1-AP)

KSHV: K8α (8C12G10G1) mouse monoclonal (Santa Cruz Biotechnology, Cat.# sc-57889), LANA (LN53) rat monoclonal (Sigma-Aldrich, Cat.# MABE1109), ORF45 (2D4A5) mouse monoclonal (Thermo Fisher, Cat.# MA5–14769), vIL6 mouse (purified in-house from v6m 12.1.1 hybridoma (ATCC) supernatant as previously described and used [54,55].

Secondary antibodies were probed for in 5% milk in TBST. Secondary antibodies used: goat anti-rabbit IgG HRP (Cell Signaling Technology, Cat.# 7074S), horse anti-mouse IgG HRP horse (Cell Signaling Technology, Cat.# 7076S), goat anti-rat IgG HRP (Invitrogen, Cat.# 31470). For each figure, immunoblots are representative of three biological replicates. Quantification was performed using ImageLab (BioRad). Quantification of the representative replicate is shown, and quantification of all three biological replicates can be found in S3 Table.

### RT-qPCR

RNA was extracted from cell pellets using the RNEasy Plus Mini Kit (Qiagen) according to the manufacturer’s protocol. RNA concentrations were measured with a NanoDrop spectrophotometer (Thermo Fisher). Equal concentrations of RNA were used to synthesize cDNA using the iScript gDNA clear cDNA synthesis kit (Bio-Rad). Gene expression was quantified by RT-qPCR in SensiFast Lo-Rox SYBR (Bioline). Ct values were normalized to β-actin and analyzed using the ΔΔCt method. The data displayed are average fold changes (2^-ΔΔCt^) between control and experimental conditions. The statistical tests are based on the ΔCt values. For two groups, an unpaired student’s t-test was used and for three or more groups, a one-way ANOVA test was used in GraphPad Prism. Primer sequences can be found in S2 Table.

### IFNβ ELISA

Supernatants were harvested and assayed by Human IFN-beta DuoSet ELISA kit (R&D Systems) according to the manufacturer’s protocol. IFNβ concentrations from supernatants were determined relative to a standard curve.

### IFNβ-luciferase assay

HEK293T were seeded 6 x 10^4^ cells per well in a 24-well plate. Twenty-four hours later they were co-transfected with firefly luciferase reporter plasmids IFNβ-luciferase (100 ng), Renilla luciferase vector (50 ng), and either an empty vector control (100 ng) or various expression plasmids (100 ng) using Lipofectamine 2000. Forty-eight hours later, the cells were harvested and luciferase activity was measured using the Dual-Luciferase Reporter Assay kit (Promega) according to the manufacturer’s protocol with a CLARIOstar plate reader. Readouts were normalized to Renilla luciferase activity. IFNβ-luciferase plasmid was kindly gifted by Dr. Zhijian Chen. Renilla luciferase plasmid (pRL-TK) was obtained from Promega. pWZL-VRK3-FLAG and pWZL-empty vector were purchased from Addgene. pUNO1-cGAS and pUNO1-IRF3 were purchased from InvivoGen. pcDNA3-STING plasmid was kindly gifted by Dr. Glen Barber. pCIG2-TBK1 plasmid was kindly gifted by Dr. Jenny Ting.

### cGAMP ELISA

Cell pellets were harvested and lysed in M-PER lysis buffer (Thermo Fisher) and assayed by 2’3’-cGAMP ELISA kit (Cayman Chemical) according to the manufacturer’s protocol. 2’3’-cGAMP concentrations from lysates were determined relative to a standard curve.

### Statistics and reproducibility

Data and error bars represent three or more independent, biological replicates, unless otherwise stated. One-way ANOVA test, student’s t-test, and multiple t-test analyses were performed when appropriate to obtain p-values using GraphPad Prism 10 software.

## Supporting information

S1 Fig Comparison of KSHV infection and reactivation using individual siRNAs versus pooled siRNA to deplete VRK3. For Fig S1A-S1B, HUVEC cells were transfected with individual siRNAs targeting VRK3 and a non-targeting control (NTC) for 24 hours, then infected with rKSHV.219 for 72 hours (n = 3). (A) At 72 hours post-infection, KSHV infection was measured by analyzing GFP^+^ cells, which were imaged by fluorescence microscopy and quantified by flow cytometry (n = 3). p-values were determined using a one-way ANOVA test and error bars indicate the standard error from the mean of three biological replicates. (B) At 72 hours post-infection, cell lysates were prepared and analyzed by immunoblot. KSHV infection was confirmed by probing for KSHV LANA and knockdowns were validated by probing for VRK3 (n = 3). For Fig S1C-S1D latently-infected iSLK.219 cells were transfected with individual siRNAs targeting VRK3 and a non-targeting control (NTC) for 48 hours, then reactivated with 50 ng/mL doxycycline for 72 hours (n = 3). (C) At 72 hours post-reactivation, cells were imaged by fluorescence microscopy for RFP and GFP signal, and the RFP^+^ cells were quantified by flow cytometry (n = 3). p-values were calculated using a one-way ANOVA test. Error bars indicate the standard error of the mean of three biological replicates. (D) Cell lysates were prepared and analyzed by immunoblot. KSHV lytic reactivation was examined by probing for KSHV K8α and knockdown was validated by probing for VRK3 (n = 3).
(TIF)

S2 Fig Localization of VRK3 in organelles within the cell. Immunofluorescence assay images of VRK3 expression in HUVEC cells with the following organelle markers: (A) AIF (mitochondria), (B) EEA1 (early endosome), (C) Lamin B1 (nuclear envelope), (D) LAMP1 (lysosome), (E) PDI (endoplasmic reticulum), and (F) RCAS1 (Golgi). (G) Immunofluorescence assays were analyzed for VRK3 and organelle marker colocalization by Pearson’s correlation coefficient (n = 3).
(TIF)

S3 Fig VRK3 knockdown increases IFNB1 transcripts. For Fig S3A-S3B, HUVEC cells were transfected with non-targeting control, VRK1, or VRK3 pooled siRNA for 96 hours. *IFNB1* mRNA transcripts were measured by RT-qPCR during the following conditions: (A) mock infection (n = 3) and (B) 72 hours post-KSHV infection (n = 3). p-values for (A) and (B) were calculated by one-way ANOVAs. Error bars indicate the standard error of the mean of three biological replicates. For Fig S3C-S3D, HUVEC cells were transfected with individual siRNAs targeted to either VRK3 or a non-targeting control (NTC) for 96 hours. IFNβ production was measured by ELISA during the following conditions: (C) mock infection (n = 3) and (D) 72 hours post-KSHV infection (n = 3). p-values for (C) and (D) were calculated using one-way ANOVA tests. Error bars indicate the standard error of the mean of three biological replicates.
(TIF)

S4 Fig VRK3 indirectly impacts STING. (A) Model of cGAS-STING signaling. (B) HEK293T cells were measured for IFNβ-luciferase activity after transfection with either an empty vector control or VRK3 expression plasmid and components of the cGAS-STING pathway (cGAS, cGAS+STING, STING, TBK1, or IRF3) in addition to an IFNβ-luciferase reporter plasmid and Renilla luciferase vector (n = 3). p-values were analyzed by student’s t-tests. Error bars indicate the standard error of the mean of three biological replicates. (C) iSLK.219 cells were transfected with pooled VRK3 siRNA or control NTC siRNA, or with BANF1 siRNA (as a positive control). At 48 hours post-transfection, cell lysates were harvested to measure 2’3’-cGAMP production by ELISA (n = 3). p-values were calculated using a one-way ANOVA test. Error bars indicate the standard error of the mean of three biological replicates. (D) HUVEC cells were depleted of VRK3 with pooled siRNA or control siRNA for 48 hours. Cell lysates were prepared and analyzed for phosphorylated STING (Ser366) and total STING protein expression by immunoblot (n = 3). (E) HUVEC cells were transfected with either NTC, VRK3, STING, or VRK3 and STING combined pooled siRNA for 48 hours. Cell lysates were prepared and analyzed for TBK1-IRF3 pathway activation by probing for phosphorylation status of TBK1 and IRF3. Knockdowns of VRK3 and STING were validated by immunoblot (n = 3).
(TIF)

S5 Fig VRK3 promotes KSHV infection in an interferon-dependent manner. (A) HUVEC cells were transfected with either non-targeting control (NTC)- or VRK3- targeted pooled siRNA and each siRNA condition was treated with either an isotype control antibody or IFNβ neutralizing antibody (10 µg/mL). Twenty-four hours later, the cells were infected with rKSHV.219. At 72 hours post-infection, KSHV infection was measured by analyzing GFP^+^ cells, which were imaged by fluorescence microscopy and quantified by flow cytometry (n = 3). p-values were calculated using a one-way ANOVA test and error bars indicate the standard error from the mean of three biological replicates. (B) HUVEC cells were transfected with NTC, VRK3, IFNB1, or VRK3 and IFNB1 combined pooled siRNA. Twenty-four hours post-transfection, cells were infected with rKSHV.219 and samples were collected 72 hours post-infection. KSHV infection was measured by analyzing GFP^+^ cells, which were imaged by fluorescence microscopy and quantified by flow cytometry (n = 3). p-values were calculated using a one-way ANOVA test and error bars indicate the standard error from the mean of three biological replicates. (C) At 72 hours post-infection, samples were collected from (A), and cell lysates were prepared and analyzed by immunoblot. KSHV infection was confirmed by probing for KSHV LANA, knockdowns were validated by probing for VRK3, and phospho-STAT1 was used as a marker for IFNβ neutralizing activity (n = 3). (D) At 72 hours post-infection, samples were collected from (B), and cell lysates were prepared and analyzed by immunoblot. KSHV infection was confirmed by probing for KSHV LANA, knockdowns were validated by probing for VRK3 and phospho-STAT1 was used as a marker for IFNB1 knockdown (n = 3).
(TIF)

S1 Raw Images Uncropped Western Blot Images. (PDF)

S1 Table siRNA sequences. (DOCX)

S2 Table RT-qPCR Primer Sequences. (DOCX)

S3 Table Western Blot Quantifications. (XLSX)

S1 Data The Source Data for the figures in the manuscript have been provided. (DOCX)
