# Supplementary figures and images for: VRK3 promotes KSHV infection by suppressing the antiviral type I interferon response
Source: PLoS Pathog. 2026 Jul 27;22(7):e1014400. doi: 10.1371/journal.ppat.1014400 (PMC13405069; doi:10.1371/journal.ppat.1014400)

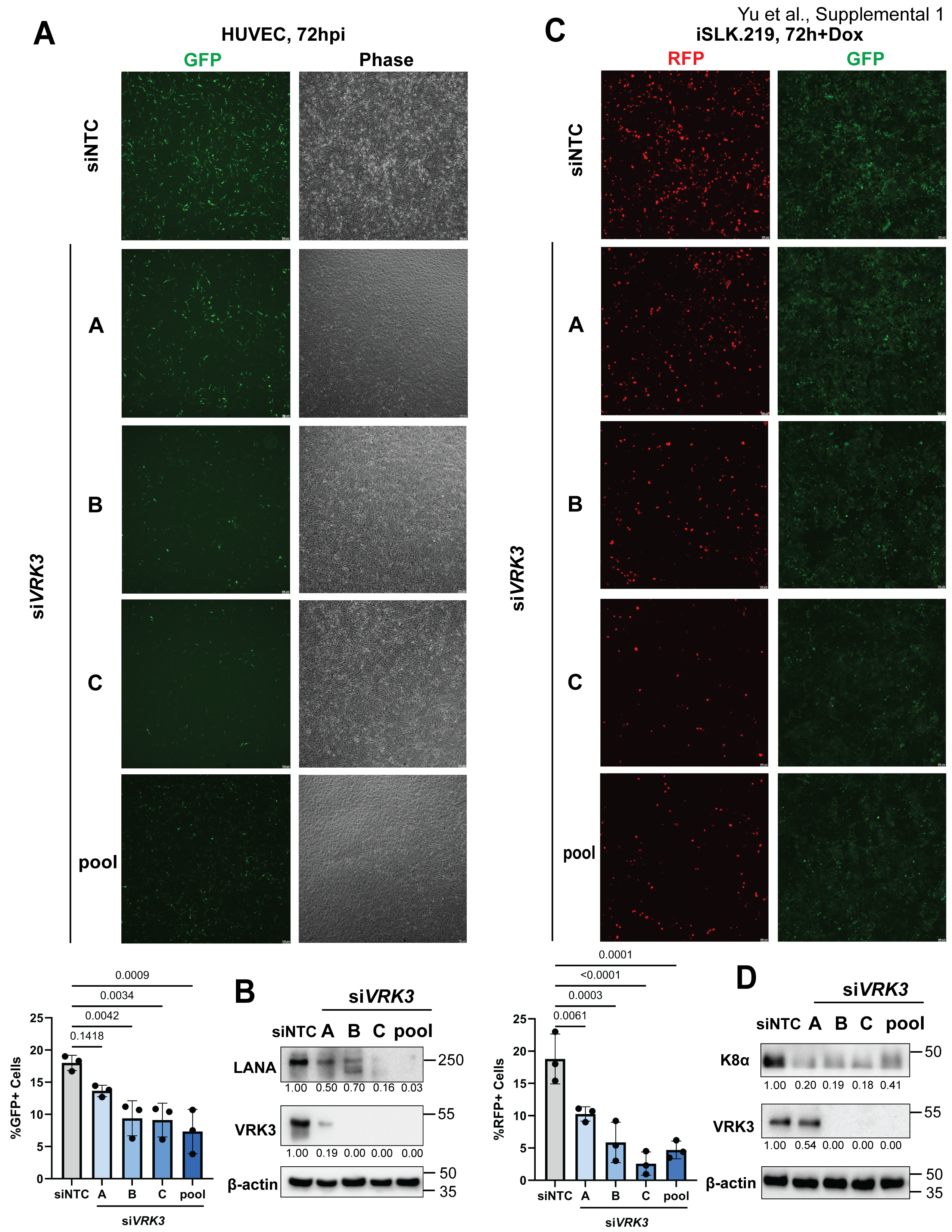

Supplement: S1 Fig — For Fig S1A-S1B, HUVEC cells were transfected with individual siRNAs targeting VRK3 and a non-targeting control (NTC) for 24 hours, then infected with rKSHV.219 for 72 hours (n = 3). (A) At 72 hours post-infection, KSHV infection was measured by analyzing GFP+ cells, which were imaged by fluorescence microscopy and quantified by flow cytometry (n = 3). p-values were determined using a one-way ANOVA test and error bars indicate the standard error from the mean of three biological replicates. (B) At 72 hours post-infection, cell lysates were prepared and analyzed by immunoblot. KSHV infection was confirmed by probing for KSHV LANA and knockdowns were validated by probing for VRK3 (n = 3). For Fig S1C-S1D latently-infected iSLK.219 cells were transfected with individual siRNAs targeting VRK3 and a non-targeting control (NTC) for 48 hours, then reactivated with 50 ng/mL doxycycline for 72 hours (n = 3). (C) At 72 hours post-reactivation, cells were imaged by fluorescence microscopy for RFP and GFP signal, and the RFP+ cells were quantified by flow cytometry (n = 3). p-values were calculated using a one-way ANOVA test. Error bars indicate the standard error of the mean of three biological replicates. (D) Cell lysates were prepared and analyzed by immunoblot. KSHV lytic reactivation was examined by probing for KSHV K8α and knockdown was validated by probing for VRK3 (n = 3). (TIF) [file ppat.1014400.s001.tif]

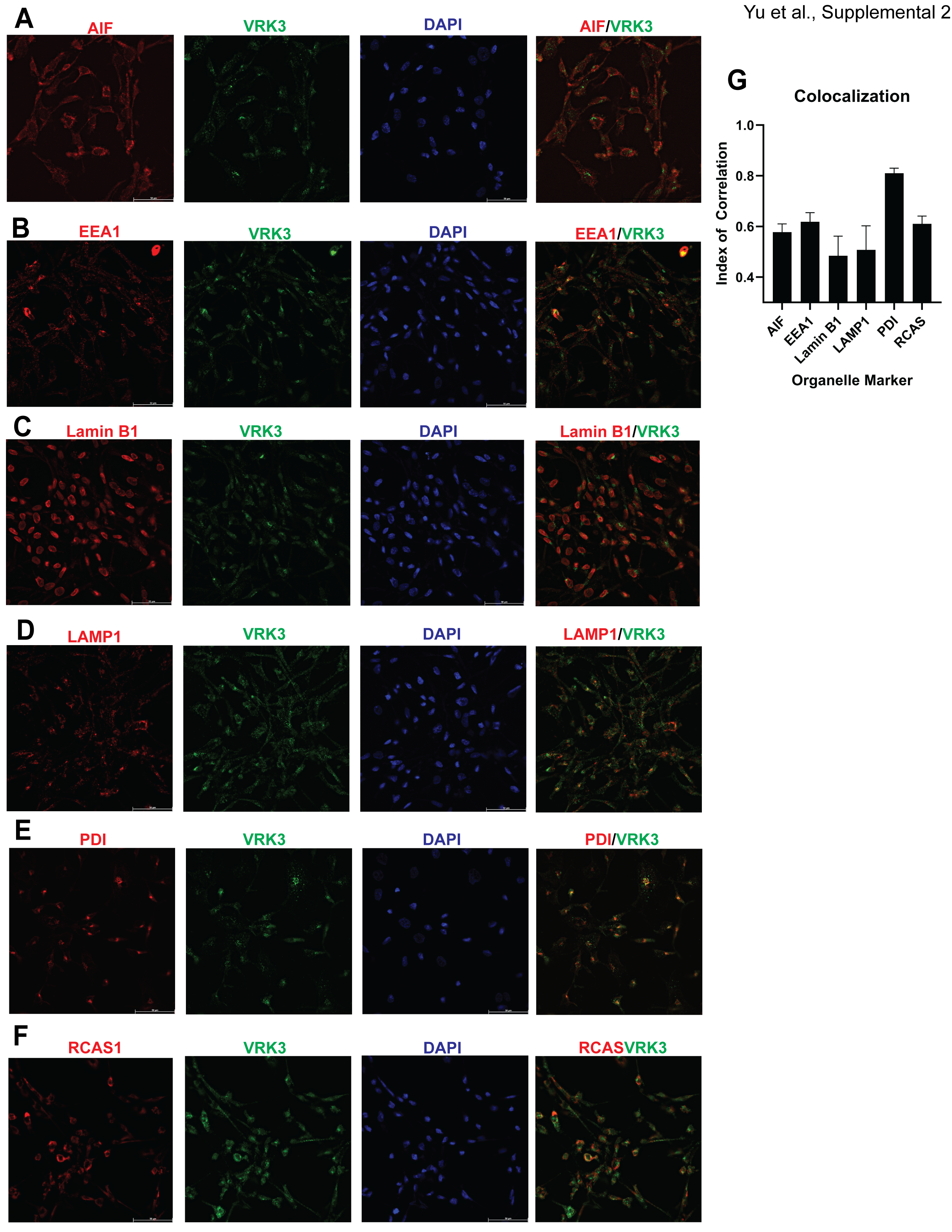

Supplement: S2 Fig — Immunofluorescence assay images of VRK3 expression in HUVEC cells with the following organelle markers: (A) AIF (mitochondria), (B) EEA1 (early endosome), (C) Lamin B1 (nuclear envelope), (D) LAMP1 (lysosome), (E) PDI (endoplasmic reticulum), and (F) RCAS1 (Golgi). (G) Immunofluorescence assays were analyzed for VRK3 and organelle marker colocalization by Pearson’s correlation coefficient (n = 3). (TIF) [file ppat.1014400.s002.tif]

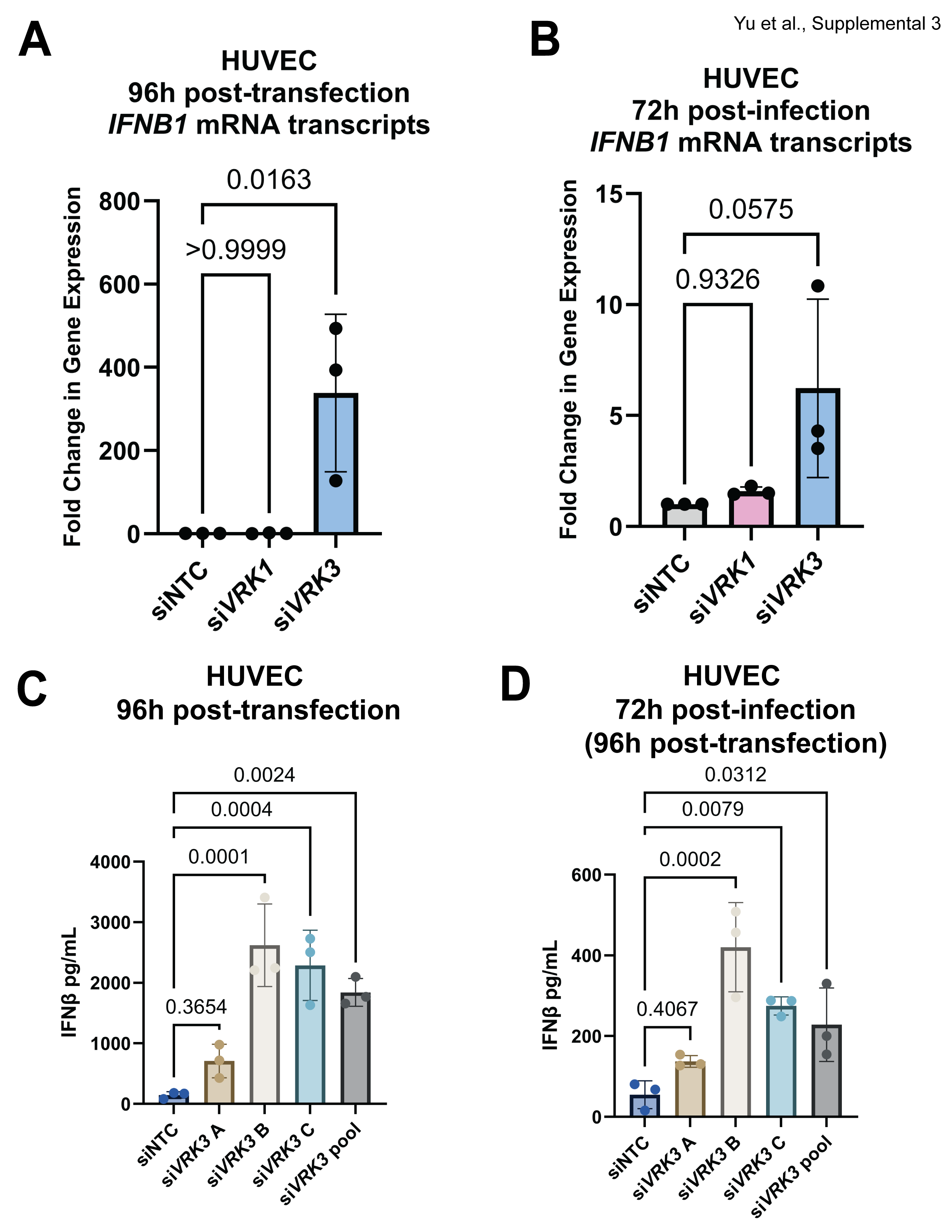

Supplement: S3 Fig — For Fig S3A-S3B, HUVEC cells were transfected with non-targeting control, VRK1, or VRK3 pooled siRNA for 96 hours. IFNB1 mRNA transcripts were measured by RT-qPCR during the following conditions: (A) mock infection (n = 3) and (B) 72 hours post-KSHV infection (n = 3). p-values for (A) and (B) were calculated by one-way ANOVAs. Error bars indicate the standard error of the mean of three biological replicates. For Fig S3C-S3D, HUVEC cells were transfected with individual siRNAs targeted to either VRK3 or a non-targeting control (NTC) for 96 hours. IFNβ production was measured by ELISA during the following conditions: (C) mock infection (n = 3) and (D) 72 hours post-KSHV infection (n = 3). p-values for (C) and (D) were calculated using one-way ANOVA tests. Error bars indicate the standard error of the mean of three biological replicates. (TIF) [file ppat.1014400.s003.tif]

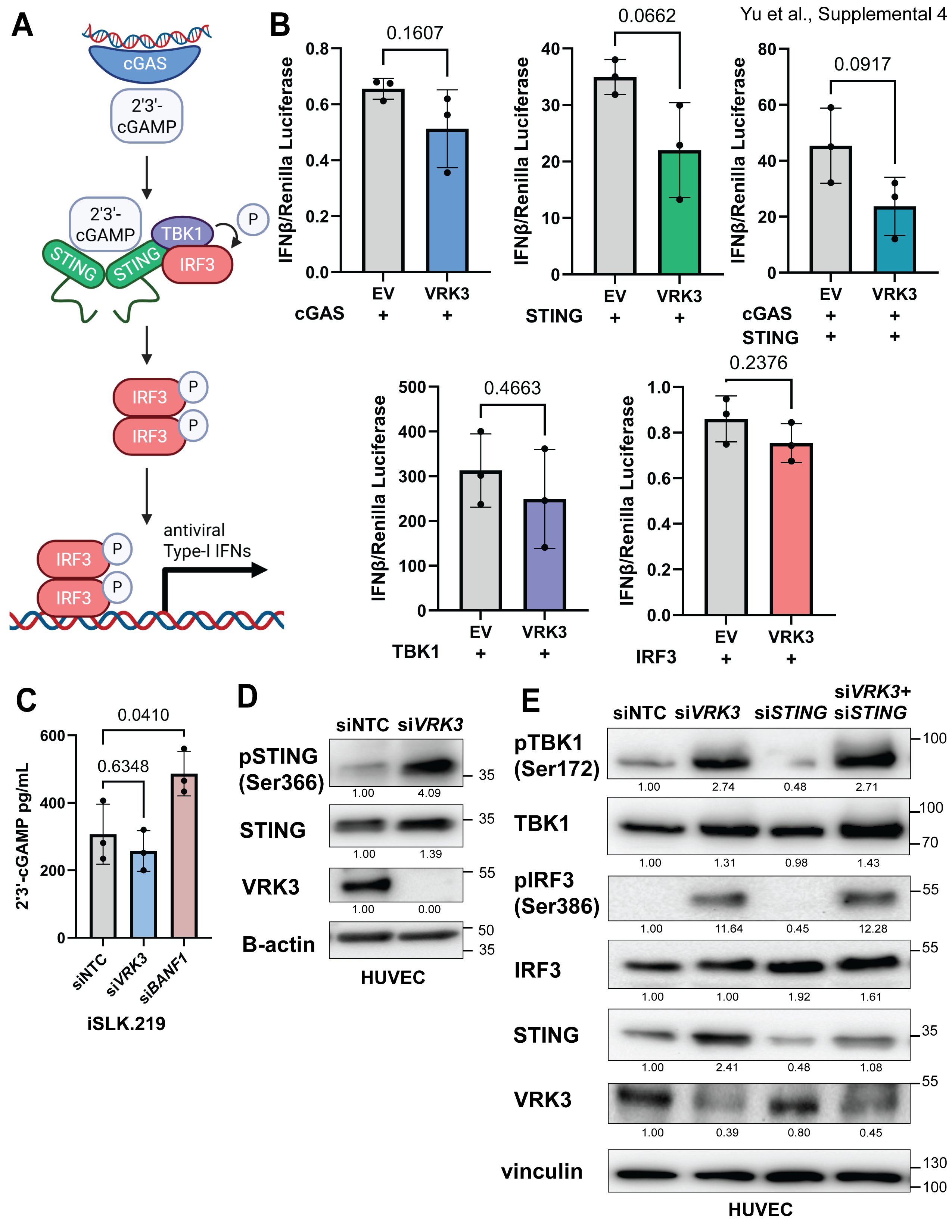

Supplement: S4 Fig — (A) Model of cGAS-STING signaling. (B) HEK293T cells were measured for IFNβ-luciferase activity after transfection with either an empty vector control or VRK3 expression plasmid and components of the cGAS-STING pathway (cGAS, cGAS+STING, STING, TBK1, or IRF3) in addition to an IFNβ-luciferase reporter plasmid and Renilla luciferase vector (n = 3). p-values were analyzed by student’s t-tests. Error bars indicate the standard error of the mean of three biological replicates. (C) iSLK.219 cells were transfected with pooled VRK3 siRNA or control NTC siRNA, or with BANF1 siRNA (as a positive control). At 48 hours post-transfection, cell lysates were harvested to measure 2’3’-cGAMP production by ELISA (n = 3). p-values were calculated using a one-way ANOVA test. Error bars indicate the standard error of the mean of three biological replicates. (D) HUVEC cells were depleted of VRK3 with pooled siRNA or control siRNA for 48 hours. Cell lysates were prepared and analyzed for phosphorylated STING (Ser366) and total STING protein expression by immunoblot (n = 3). (E) HUVEC cells were transfected with either NTC, VRK3, STING, or VRK3 and STING combined pooled siRNA for 48 hours. Cell lysates were prepared and analyzed for TBK1-IRF3 pathway activation by probing for phosphorylation status of TBK1 and IRF3. Knockdowns of VRK3 and STING were validated by immunoblot (n = 3). (TIF) [file ppat.1014400.s004.tif]

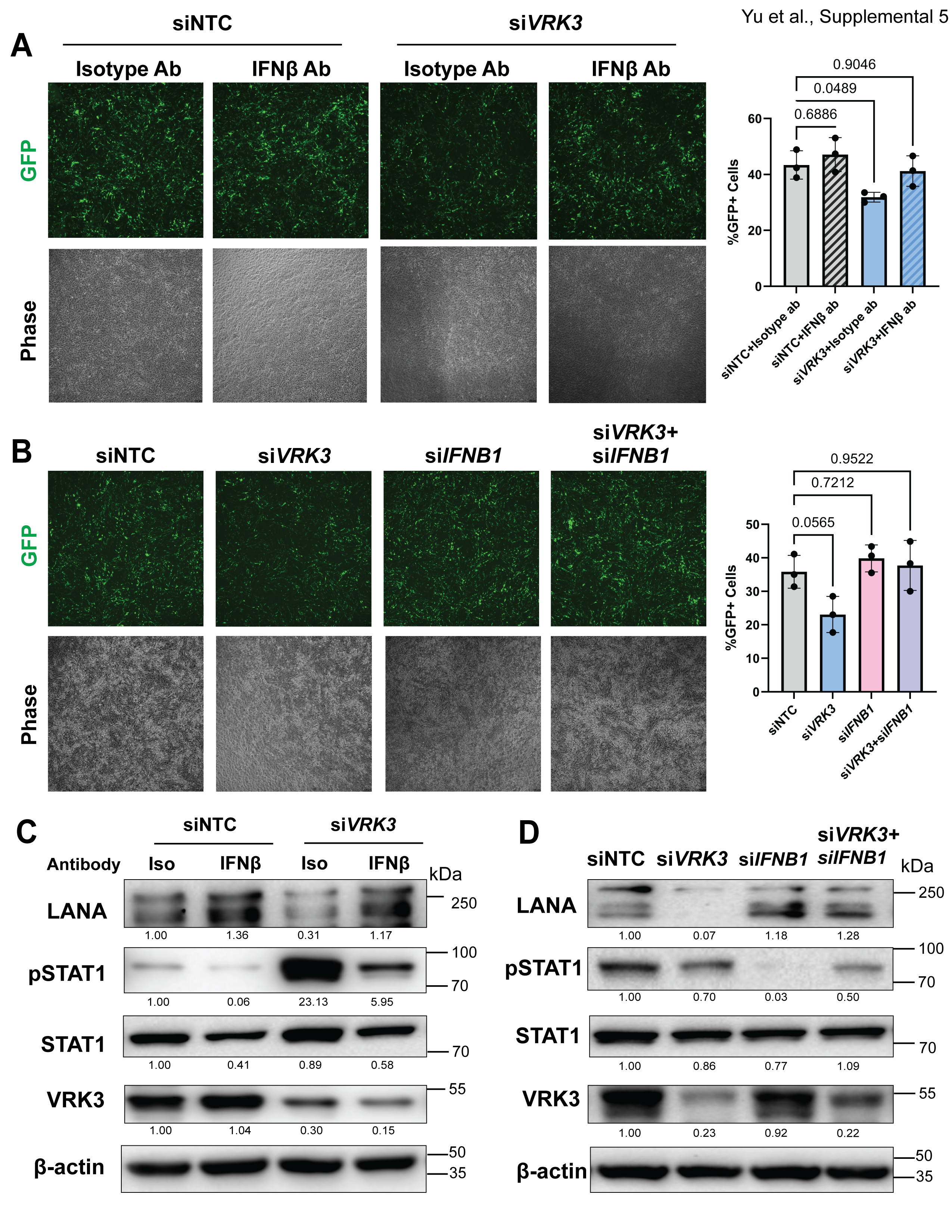

Supplement: S5 Fig — (A) HUVEC cells were transfected with either non-targeting control (NTC)- or VRK3- targeted pooled siRNA and each siRNA condition was treated with either an isotype control antibody or IFNβ neutralizing antibody (10 µg/mL). Twenty-four hours later, the cells were infected with rKSHV.219. At 72 hours post-infection, KSHV infection was measured by analyzing GFP+ cells, which were imaged by fluorescence microscopy and quantified by flow cytometry (n = 3). p-values were calculated using a one-way ANOVA test and error bars indicate the standard error from the mean of three biological replicates. (B) HUVEC cells were transfected with NTC, VRK3, IFNB1, or VRK3 and IFNB1 combined pooled siRNA. Twenty-four hours post-transfection, cells were infected with rKSHV.219 and samples were collected 72 hours post-infection. KSHV infection was measured by analyzing GFP+ cells, which were imaged by fluorescence microscopy and quantified by flow cytometry (n = 3). p-values were calculated using a one-way ANOVA test and error bars indicate the standard error from the mean of three biological replicates. (C) At 72 hours post-infection, samples were collected from (A), and cell lysates were prepared and analyzed by immunoblot. KSHV infection was confirmed by probing for KSHV LANA, knockdowns were validated by probing for VRK3, and phospho-STAT1 was used as a marker for IFNβ neutralizing activity (n = 3). (D) At 72 hours post-infection, samples were collected from (B), and cell lysates were prepared and analyzed by immunoblot. KSHV infection was confirmed by probing for KSHV LANA, knockdowns were validated by probing for VRK3 and phospho-STAT1 was used as a marker for IFNB1 knockdown (n = 3). (TIF) [file ppat.1014400.s005.tif]
