# Supplementary material for: VRK3 promotes KSHV infection by suppressing the antiviral type I interferon response
Source: PLoS Pathog. 2026 Jul 27;22(7):e1014400. doi: 10.1371/journal.ppat.1014400 (PMC13405069; doi:10.1371/journal.ppat.1014400)

Figure 1A

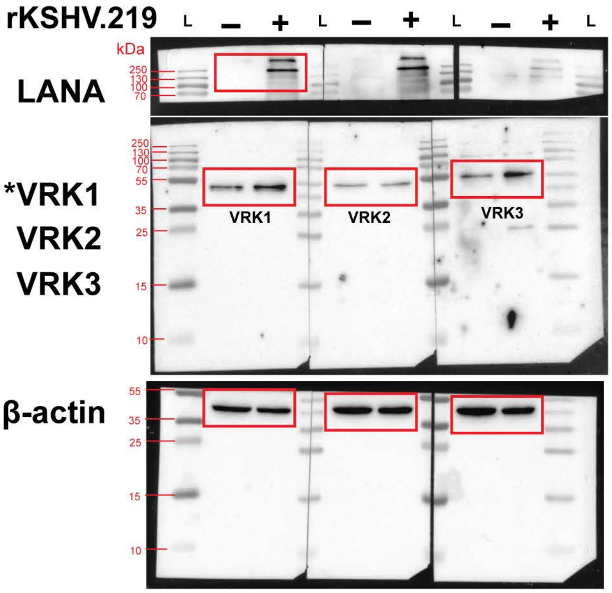

*ran in triplicate from 1 experiment to avoid stripping;
VRKs all around same molecular weight

Figure 1D

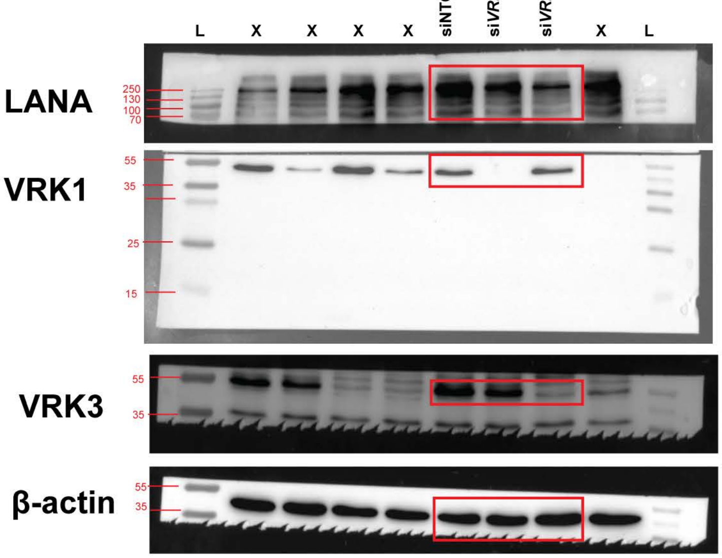

Figure 1F

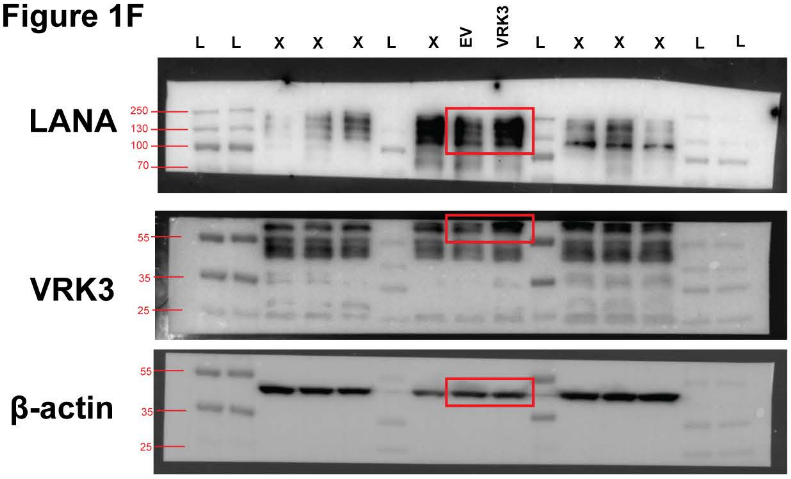

Figure 2B

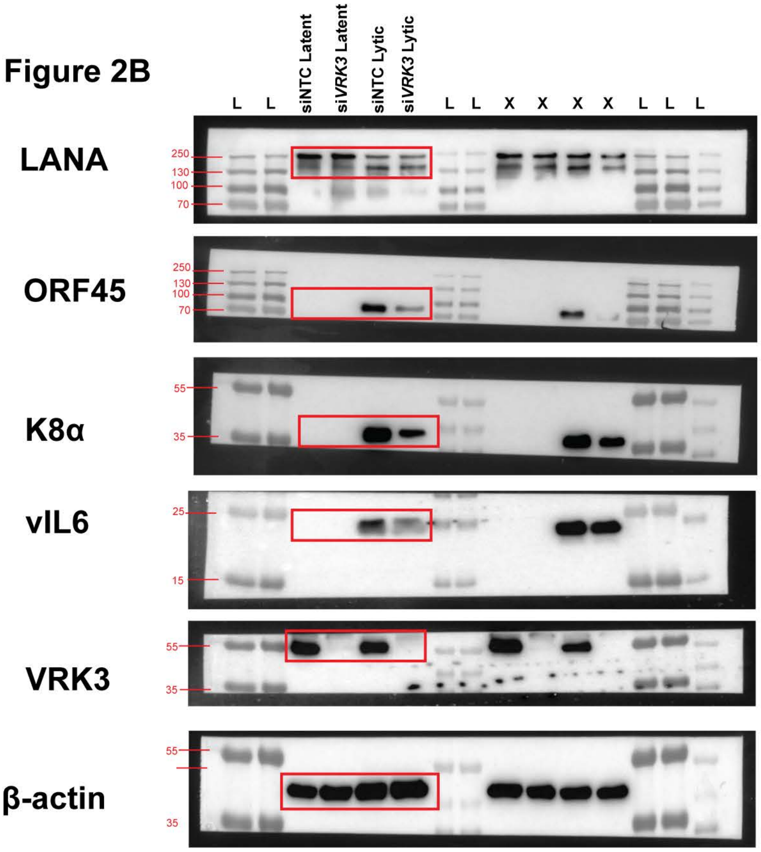

Figure 2H

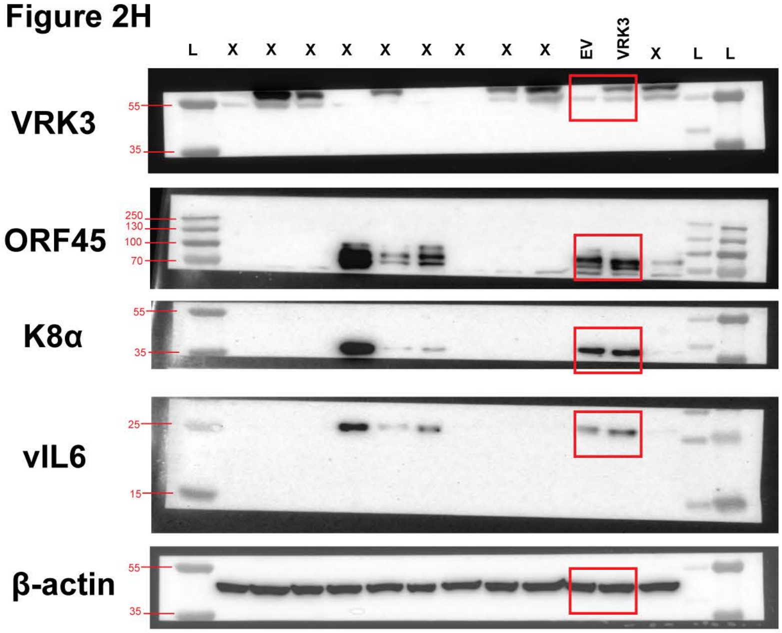

L=Molecular Weight Ladder;
red box is cropped area used in indicated Figure

Figure 3C

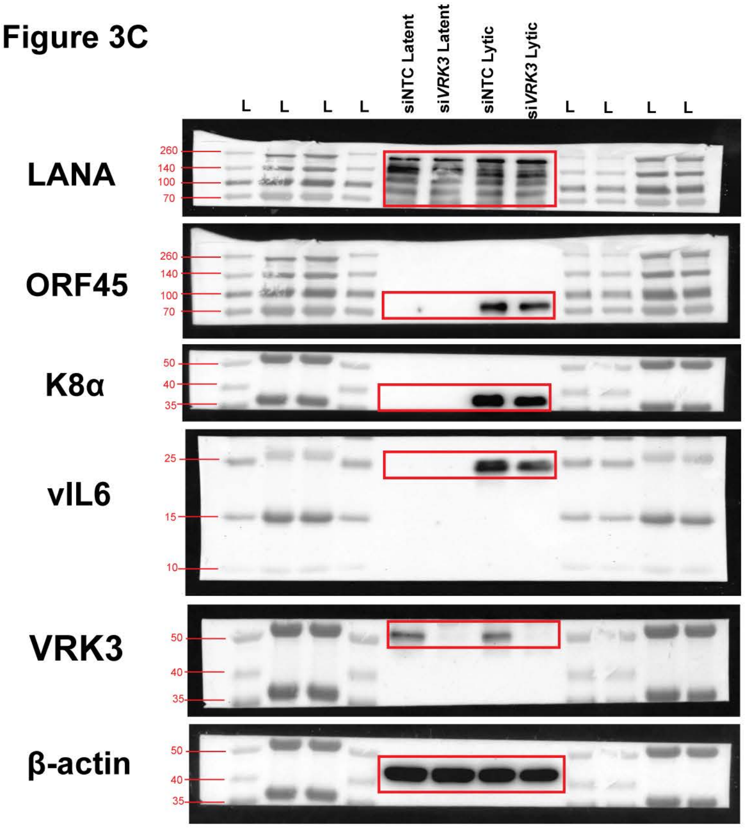

Figure 4A

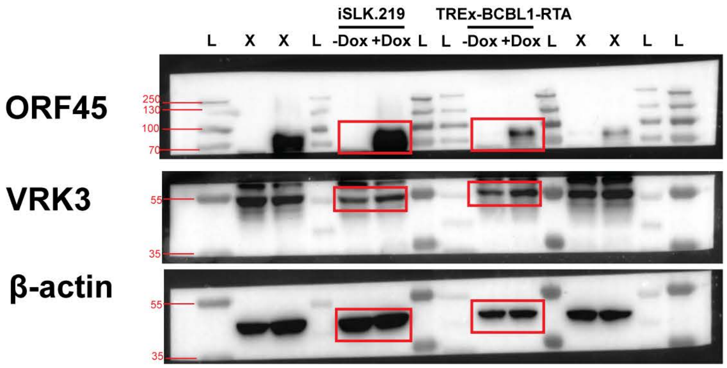

Figure 6A

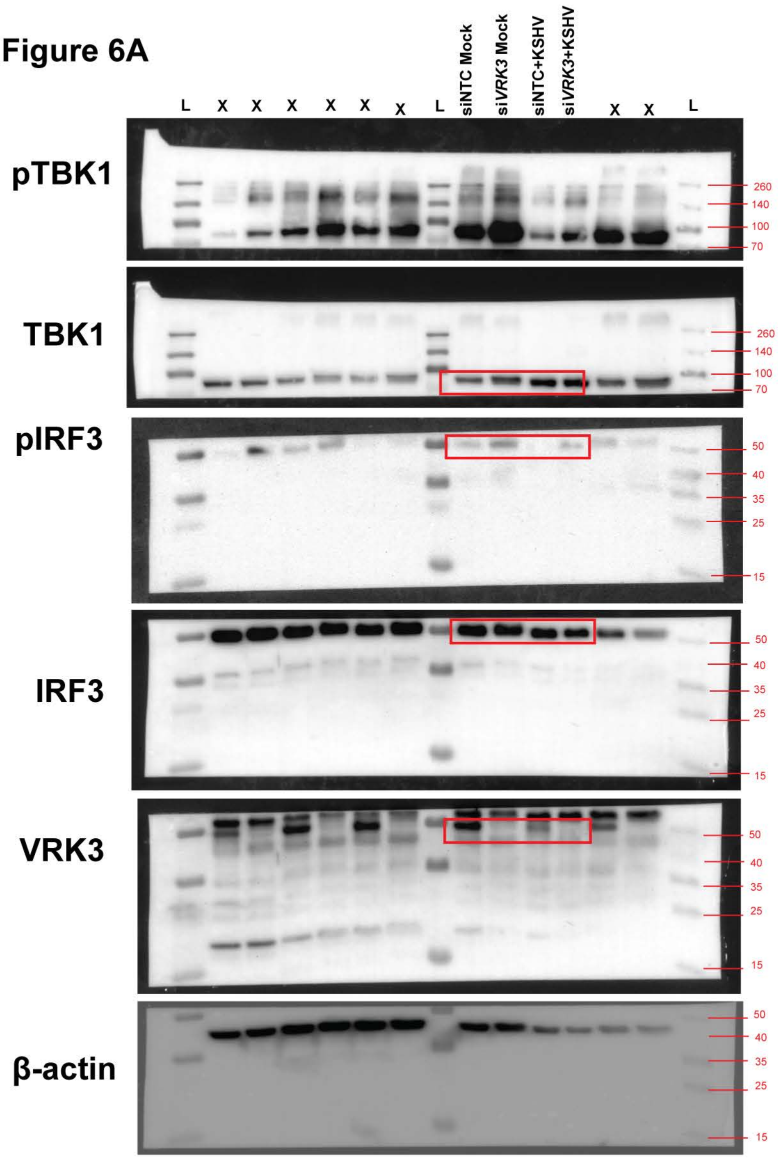

Figure 6B

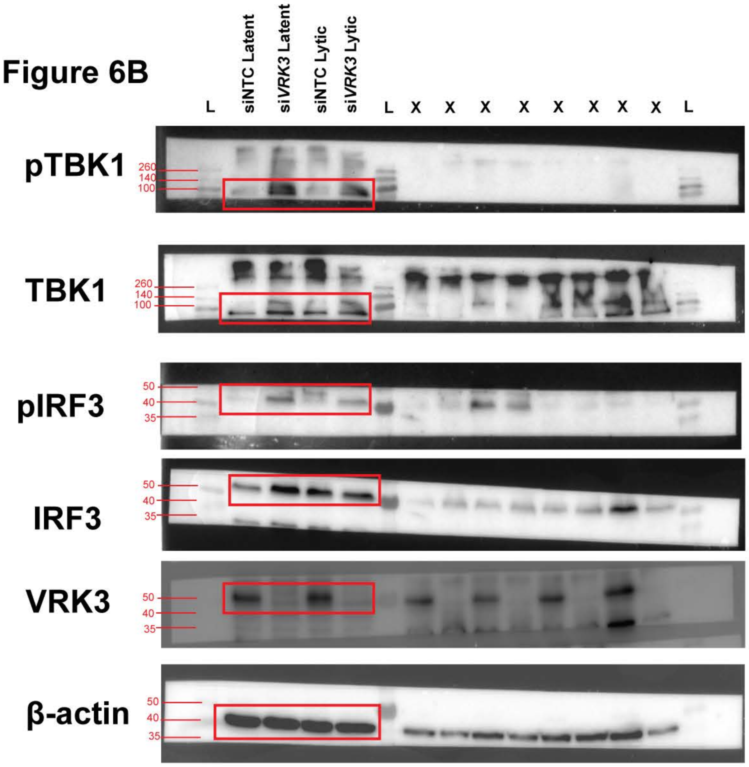

Figure 6C

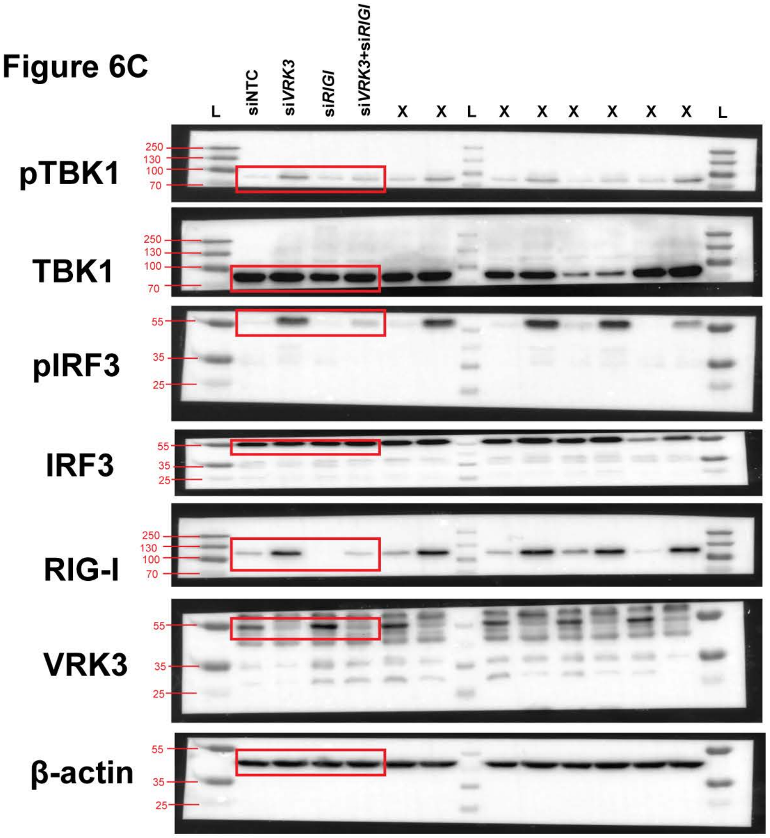

Figure 6E

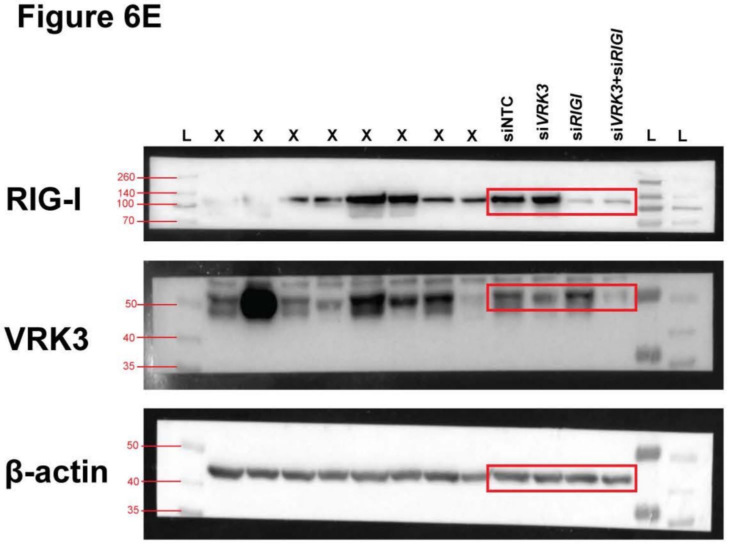

Figure S1B

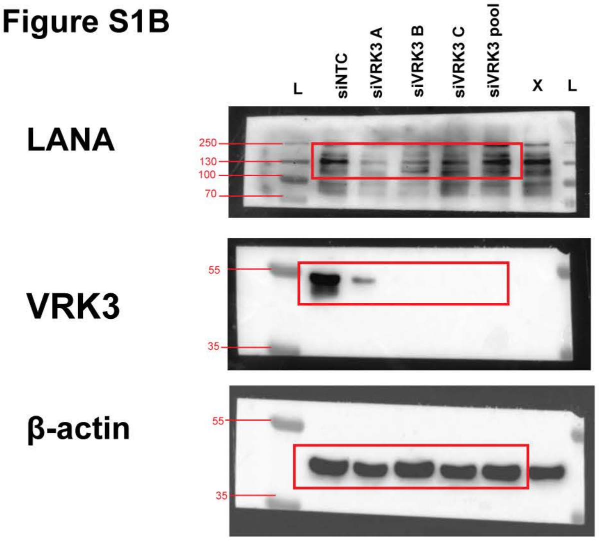

Figure S1D

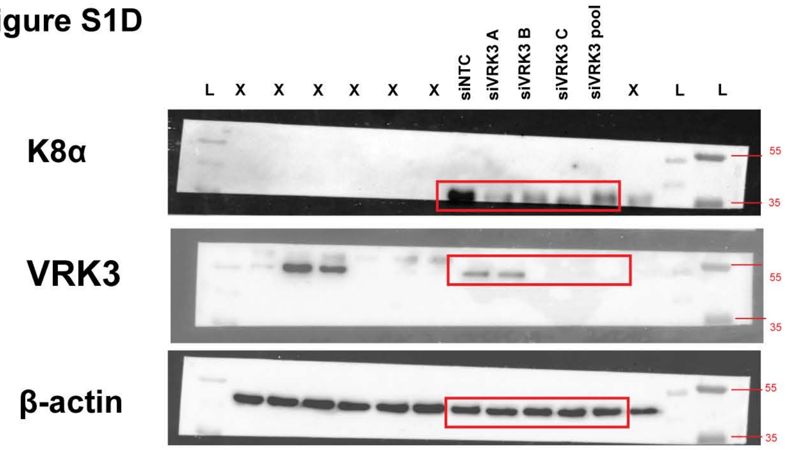

Figure S4D

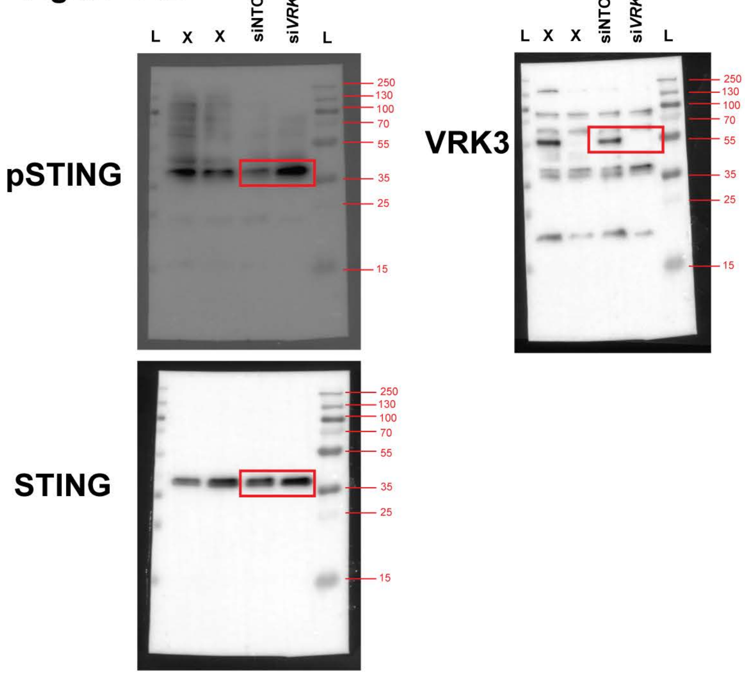

Figure S4E

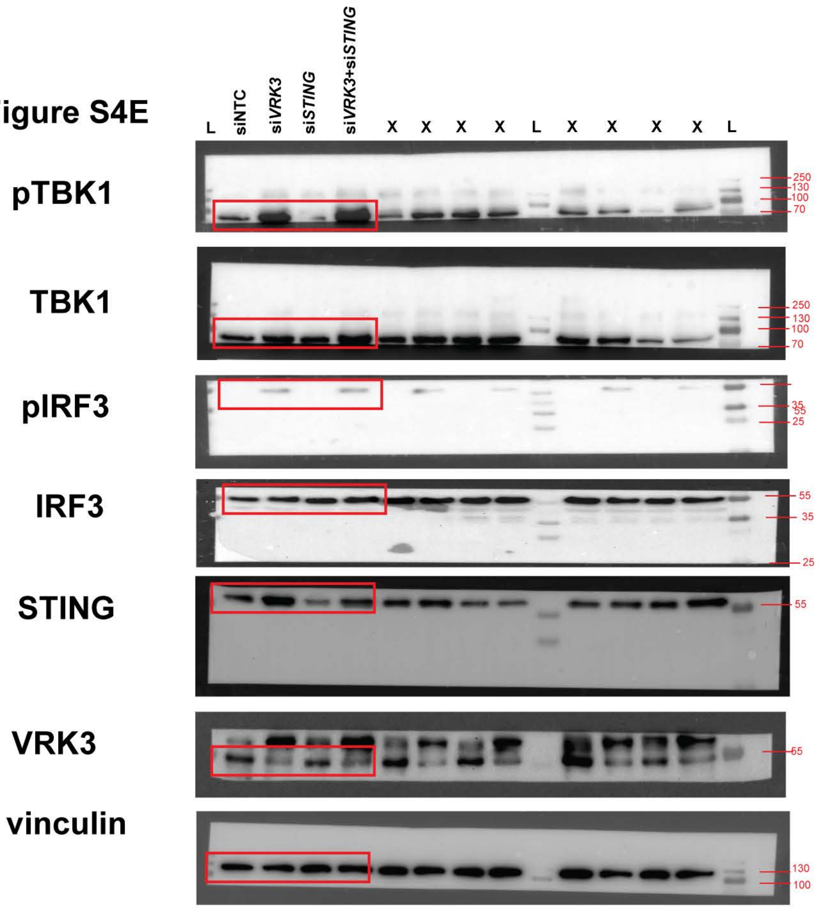

Figure S5C

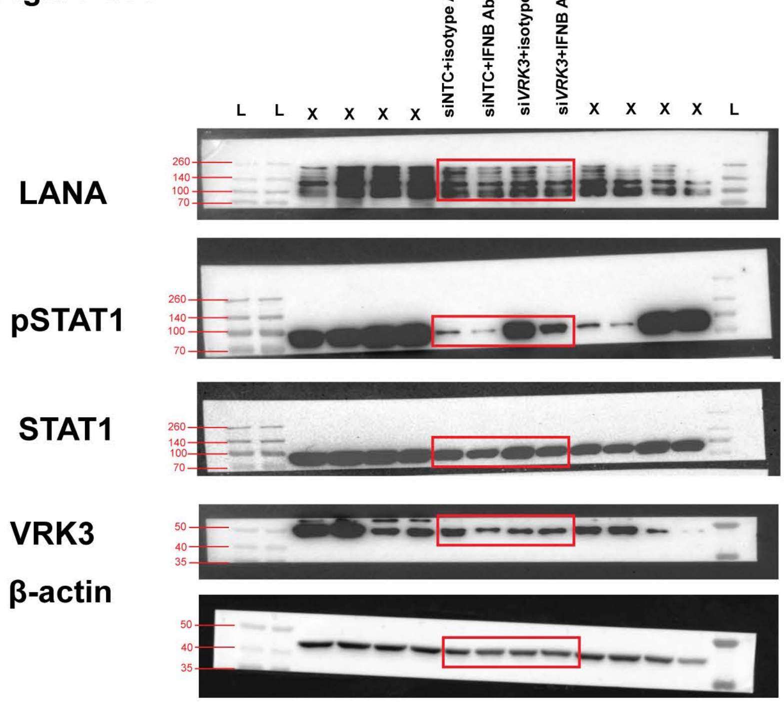

Figure S5D

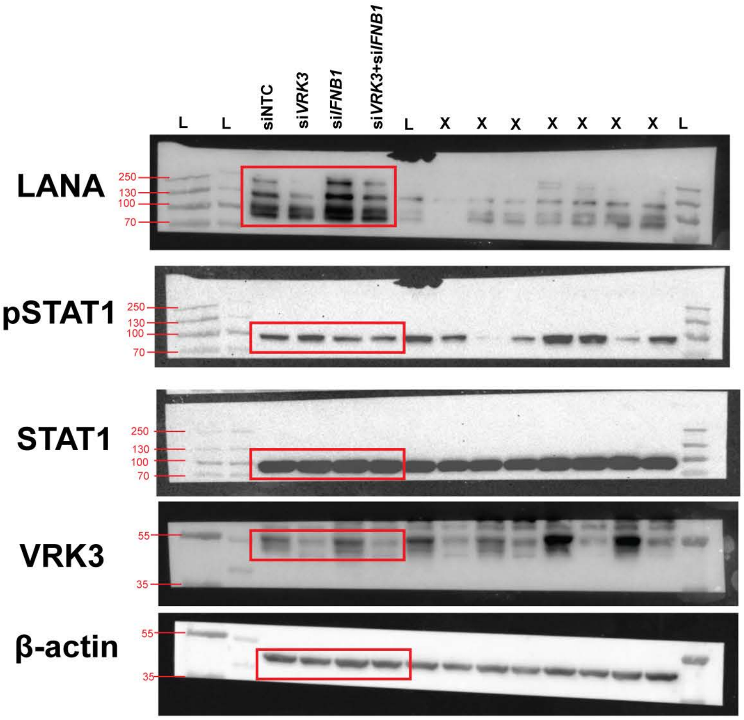

Supplement: S1 Raw Images — (PDF) [file ppat.1014400.s006.pdf]
