# Supplementary material for: VRK3 promotes KSHV infection by suppressing the antiviral type I interferon response
Source: PLoS Pathog. 2026 Jul 27;22(7):e1014400. doi: 10.1371/journal.ppat.1014400 (PMC13405069; doi:10.1371/journal.ppat.1014400)
Supplement: S1 Table — (DOCX) [file ppat.1014400.s007.docx]

**Table S1. siRNA Sequences**

| **Target Gene** | **Sequences** |
| --- | --- |
| *BANF1* SMARTpool | AAAGAUUGCUAUUGUCGUA,  GGUGCUAAAGAAAGAUGAA,  GCACCGAGACUUCGUGGCA,  UUGGUGAAGUCCUGGGCAA |
| *IFNB1* SMARTpool | GGAAUGAGACUAUUGUUGA,  AUGGGAGGAUUCUGCAUUA,  CAACUUGCUUGGAUUCCUA,  GCAUUGACCAUCUAUGAGA |
| NTC SMARTpool | UGGUUUACAUGUCGACUAA,  UGGUUUACAUGUUGUGUGA,  UGGUUUACAUGUUUUCUGA,  UGGUUUACAUGUUUUCCUA |
| NTC individual | UGGUUUACAUGUCGACUAA |
| *RIG-I* SMARTpool | GCACAGAAGUGUAUAUAUUG,  CCACAACACUAGUAAACAA,  CGGAUUAGCGACAAAUUUA,  UCGAUGAGAUUGAGCAAGA |
| *STING* SMARTpool | GGUCAUAUUACAUCGGAUA,  AACAUUCGCUUCCUGGAUA,  GCAUCAAGGAUCGGGUUUA,  GCACCUGUGUCCUGGAGUA |
| *VRK1* SMARTpool | GCAGUUGGAGAGAUAAUAA,  AUACUUGGUUAUUGCAUGA,  GGCUUUGGCUGUAUAUAUC,  AGGUGUACUUGGUAGAUUA |
| *VRK3* SMARTpool | CCAGCGGCAUUCAAAUUC,  CUGCAUGGUUUCGGUGUU,  GGACAGUGCUGACAGACAA,  CCACCUCACUUGAAGCUUU |
| *VRK3* individual A | CCAGCGGCAUUCAAAUUC |
| *VRK3* individual B | CUGCAUGGUUUCGGUGUU |
| *VRK3* individual C | CCACCUCACUUGAAGCUUU |
