# Supplementary material for: VRK3 promotes KSHV infection by suppressing the antiviral type I interferon response
Source: PLoS Pathog. 2026 Jul 27;22(7):e1014400. doi: 10.1371/journal.ppat.1014400 (PMC13405069; doi:10.1371/journal.ppat.1014400)
Supplement: S2 Table — (DOCX) [file ppat.1014400.s008.docx]

**Table S2. RT-qPCR Primer Sequences**

| **RT-qPCR** | **Forward Primer 5’-3’** | **Reverse Primer 5’-3’** |
| --- | --- | --- |
| *ACTIN* | ACCTTCTACAATGAGCTGCG | CCTGGATAGCAACGTACATGG |
| *VRK1* | TGGCAAATTGGACCTCAGTG | TGGTTCTTGAACGGGTCTGT |
| *VRK3* | CTATTGCCCAAGTGGCAAAC | CTCAGTGTTGGGAAGGCAAT |
| *IFNB1* | GTTGAGAACCTCCTGGCTAATG | GGTAATGCAGAATCCTCCCATAATA |
| *OAS1* | GCGCCCCACCAAGCTCAAGA | GCTCCCTCGCTCCCAAGCAT |
| *OAS2* | ACCCGAACAGTTCCCCCTGGT | ACAAGGGTACCATCGGAGTTGCC |
| *IFIT2* | GGGAAACTATGCCTGGGTC | CCTTCGCTCTTTCATATTTGGTTTC |
| *KSHV LANA* | CGGAGCTAAAGAGTCTGGTG | GCAGTCTCCAGAGTCTTCTC |
| *KSHV vIL6* | CGGTTCACTGCTGGTATCTG | CAGTATCGTTGATGGCTGGT |
| *KSHV K8.1* | AAAGCGTCCAGGCCACCACAGA | GGCAGAAAATGGCACACGGTTAC |
| *KSHV ORF57* | TGGACATTATGAAGGGCATCCTA | CGGGTTCGGACAATTGCT |
| *KSHV ORF39* | GGTTTCCCCTGCTACTTCAA | CATGCTTGGCCCGATATAC |
