# Supplementary material for: VRK3 promotes KSHV infection by suppressing the antiviral type I interferon response
Source: PLoS Pathog. 2026 Jul 27;22(7):e1014400. doi: 10.1371/journal.ppat.1014400 (PMC13405069; doi:10.1371/journal.ppat.1014400)
Supplement: S1 Data — (DOCX) [file ppat.1014400.s010.docx]

| **1B** |  |  |  |  |
| --- | --- | --- | --- | --- |
| Flow Cytometry | %GFP+ Cells | siNTC | siVRK1 | siVRK3 |
|  |  | 33.3 | 38.1 | 20.7 |
|  |  | 41.7 | 42.9 | 21.65 |
|  |  | 42.85 | 41.7 | 25.55 |

| **1C** |  |  |  |  |  |  |  |  |  |
| --- | --- | --- | --- | --- | --- | --- | --- | --- | --- |
| RTqPCR | Fold Change | siNTC | siVRK1 | siVRK3 |  | dCt | siNTC | siVRK1 | siVRK3 |
|  | VRK1 | 1 | 0.072118 | 4.274599 |  |  | 6.214 | 10.008 | 4.118 |
|  |  | 1 | 0.029503 | 3.487545 |  |  | 6.188 | 11.271 | 4.386 |
|  |  | 1 | 0.0968 | 0.8914 |  |  | 5.503 | 8.871 | 5.668 |
|  | VRK3 | siNTC | siVRK1 | siVRK3 |  | dCt | siNTC | siVRK1 | siVRK3 |
|  |  | 1 | 1.421648 | 0.102721 |  |  | 6.141 | 5.633 | 9.424 |
|  |  | 1 | 0.694372 | 0.09997 |  |  | 6.032 | 6.558 | 9.354 |
|  |  | 1 | 0.4206 | 0.028 |  |  | 6.866 | 8.116 | 12.024 |

| **1E** |  |  |  |
| --- | --- | --- | --- |
| Flow Cytometry | %GFP+ Cells | EV | VRK3 |
|  |  | 15.1 | 41.8 |
|  |  | 17.9 | 40.0 |
|  |  | 28.7 | 45.7 |

| **2A** |  |  |  |
| --- | --- | --- | --- |
| Flow Cytometry | %RFP+ Cells | siNTC | siVRK3 |
|  |  | 14.5 | 5.71 |
|  |  | 17.5 | 6.38 |
|  |  | 22.9 | 8.46 |

| **2C** |  |  |  |  |  |  |  |
| --- | --- | --- | --- | --- | --- | --- | --- |
| RTqPCR | Fold Change | siNTC | siVRK3 |  | dCt | siNTC | siVRK3 |
|  | vIL6 | 1 | 0.440899 |  | vIL6 | -4.78 | -3.598 |
|  |  | 1 | 0.0899 |  |  | -6.221 | -2.746 |
|  |  | 1 | 0.470957 |  |  | -4.73192 | -3.64559 |
|  |  | siNTC | siVRK3 |  |  | siNTC | siVRK3 |
|  | K8.1 | 1 | 0.227935 |  | K8.1 | -0.882 | 1.251 |
|  |  | 1 | 0.0408 |  |  | -2.507 | 2.109 |
|  |  | 1 | 0.181515 |  |  | -2.35449 | 0.107348 |
|  |  | siNTC | siVRK3 |  |  | siNTC | siVRK3 |
|  | ORF57 | 1 | 0.354962 |  | ORF57 | -1.98 | -0.485 |
|  |  | 1 | 0.1144 |  |  | -2.391 | 0.737 |
|  |  | 1 | 0.205711 |  |  | -1.16966 | 1.111652 |

| **2D** |  |  |  |  |  |  |  |
| --- | --- | --- | --- | --- | --- | --- | --- |
| RTqPCR | Fold Change | siNTC | siVRK3 |  | dCt | siNTC | siVRK3 |
|  | Intracellular Genomes | 1 | 0.567949 |  | Intracellular Genomes | 1.594 | 2.41 |
|  |  | 1 | 0.27409 |  |  | 2.189 | 4.057 |
|  |  | 1 | 0.272127 |  |  | 1.715 | 3.593 |

| **2E** |  |  |  |
| --- | --- | --- | --- |
| RTqPCR | Viral Genomes in Supernatant | siNTC | siVRK3 |
|  |  | 4628953 | 490003 |
|  |  | 1819992 | 230157 |
|  |  | 3712497 | 79573 |

| **2G** |  |  |  |
| --- | --- | --- | --- |
| Flow Cytometry | %GFP+ Cells | siNTC | siVRK3 |
|  |  | 11.4 | 6.81 |
|  |  | 11.5 | 7.37 |
|  |  | 15.4 | 7.97 |

| **3A** |  |  |  |  |  |  |  |
| --- | --- | --- | --- | --- | --- | --- | --- |
| RTqPCR | Fold Change | siNTC | siVRK3 |  | dCt | siNTC | siVRK3 |
|  | VRK3 | 1 | 0.114769 |  | VRK3 | 6.292 | 9.415 |
|  |  | 1 | 0.151596 |  |  | 5.784 | 8.505 |
|  |  | 1 | 0.240115 |  |  | 6.317 | 8.375 |

| **3B** |  |  |  |  |  |  |  |
| --- | --- | --- | --- | --- | --- | --- | --- |
| RTqPCR | Fold Change | siNTC | siVRK3 |  | dCt | siNTC | siVRK3 |
|  | vIL6 | 1 | 0.593457 |  | vIL6 | -1.682 | -0.774 |
|  |  | 1 | 0.151781 |  |  | -2.865 | -0.145 |
|  |  | 1 | 0.408014 |  |  | -2.504 | -1.21 |
|  |  | siNTC | siVRK3 |  |  | siNTC | siVRK3 |
|  | K8.1 | 1 | 0.068726 |  | K8.1 | 1.133 | 4.996 |
|  |  | 1 | 0.340111 |  |  | 1 | 2.556 |
|  |  | 1 | 0.299587 |  |  | 0.768 | 2.507 |
|  |  | siNTC | siVRK3 |  |  | siNTC | siVRK3 |
|  | ORF57 | 1 | 0.468461 |  | ORF57 | 0.262 | 1.356 |
|  |  | 1 | 0.651459 |  |  | 0.285 | 1.177 |
|  |  | 1 | 0.538777 |  |  | 0.777 | 1.277 |

| **3D** |  |  |  |
| --- | --- | --- | --- |
| RTqPCR | Viral Genomes in Supernatant | siNTC | siVRK3 |
|  |  | 2205122 | 930254.2 |
|  |  | 2254264 | 998496 |
|  |  | 2938915 | 1421513 |

| **4B** |  |  |  |  |  |  |
| --- | --- | --- | --- | --- | --- | --- |
| IFA | Mean VRK3 Intensity % | | SLK | | iSLK.219 | |
|  |  |  | Mean | SEM | Mean | SEM |
|  |  | Cytoplasmic | 29.00000 | 3.17346 | 40.63343 | 4.46315 |
|  |  | Perinuclear | 22.77662 | 2.94397 | 22.31075 | 2.59016 |
|  |  | Nuclear | 48.17023 | 4.70677 | 37.05582 | 4.06453 |

| **4C** |  |  |  |  |  |  |
| --- | --- | --- | --- | --- | --- | --- |
| IFA | Mean VRK3 Intensity % | | Latent | | Lytic | |
|  |  |  | Mean | SEM | Mean | SEM |
|  |  | Cytoplasmic | 40.63343 | 4.46315 | 28.52993 | 8.724139 |
|  |  | Perinuclear | 22.31075 | 2.59016 | 10.26356 | 4.169281 |
|  |  | Nuclear | 37.05582 | 4.06453 | 61.2065 | 10.07618 |

| **4D** |  |  |  |
| --- | --- | --- | --- |
| IFA | Number of Puncta | Latent | Lytic |
|  |  | 2 | 72 |
|  |  | 5 | 24 |
|  |  | 4 | 71 |
|  |  | 10 | 19 |
|  |  | 11 | 60 |
|  |  | 7 | 112 |
|  |  | 0 | 51 |
|  |  | 15 | 12 |
|  |  | 5 | 32 |
|  |  | 6 | 75 |
|  |  | 5 | 56 |
|  |  | 9 | 19 |
|  |  | 0 | 61 |
|  |  | 16 | 68 |
|  |  | 2 | 140 |
|  |  | 1 | 39 |
|  |  | 9 | 129 |
|  |  | 10 | 45 |
|  |  | 2 | 70 |
|  |  | 9 | 97 |
|  |  | 0 | 98 |
|  |  | 9 | 51 |
|  |  | 15 | 30 |
|  |  | 6 | 36 |
|  |  | 0 | 10 |
|  |  | 8 | 164 |
|  |  | 21 | 168 |
|  |  | 15 | 24 |
|  |  | 3 | 29 |
|  |  | 7 | 74 |
|  |  | 0 |  |
|  |  | 0 |  |
|  |  | 11 |  |
|  |  | 11 |  |
|  |  | 8 |  |
|  |  | 10 |  |

| **4E** |  |  |  |  |
| --- | --- | --- | --- | --- |
| IFA | Radial Peak Distance um | | Latent | Lytic |
|  |  |  | 0.60666 | 4.152908 |
|  |  |  | 1.902079 | 16.04857 |
|  |  |  | 5.23893 | 2.991379 |
|  |  |  | 3.702647 | 4.768716 |
|  |  |  | 1.605098 | 3.979152 |
|  |  |  | 4.079167 | 4.373398 |
|  |  |  | 0.866975 | 2.86832 |
|  |  |  | 2.796318 | 0.266904 |
|  |  |  | 4.289915 | 0.33363 |
|  |  |  | 4.460218 | 3.467132 |
|  |  |  | 2.568211 | 14.19185 |
|  |  |  | 17.85158 | 0.60969 |
|  |  |  | 3.137552 | 4.458494 |
|  |  |  | 0.803397 | 17.10903 |
|  |  |  | 1.712141 | 4.312481 |
|  |  |  | 4.392485 | 10.96184 |
|  |  |  | 2.990924 | 7.821342 |
|  |  |  | 1.356709 | 16.95737 |
|  |  |  | 1.164105 | 2.022579 |
|  |  |  | 8.170004 | 5.605125 |
|  |  |  | 1.315133 | 4.738014 |
|  |  |  | 9.609095 | 3.221737 |
|  |  |  | 5.916768 | 3.281073 |
|  |  |  | 3.241913 | 18.63721 |
|  |  |  | 2.648977 | 6.325257 |
|  |  |  | 3.407244 | 26.02929 |
|  |  |  | 1.669901 |  |
|  |  |  | 2.132982 |  |
|  |  |  | 4.313647 |  |
|  |  |  | 2.126033 |  |
|  |  |  | 5.697806 |  |
|  |  |  | 1.555374 |  |
|  |  |  | 1.749433 |  |
|  |  |  | 3.993615 |  |

| **5A** |  |  |  |  |  |  |  |
| --- | --- | --- | --- | --- | --- | --- | --- |
| RTqPCR | Fold Change | siNTC 72hpt | siVRK3 72hpt |  | dCt | siNTC 72hpt | siVRK3 72hpt |
|  | IFNB1 | 1 | 206.3235 |  | IFNB1 | 15.19998 | 7.511215 |
|  |  | 1 | 281.887 |  |  | 17.512 | 9.373 |
|  |  | 1 | 129.7164 |  |  | 13.79 | 6.771 |
|  |  |  |  |  |  |  |  |
|  |  | siNTC 96hpt | siVRK3 96hpt |  | dCt | siNTC 96hpt | siVRK3 96hpt |
|  | IFNB1 | 1 | 493.9572 |  | IFNB1 | 7.187 | -1.762 |
|  |  | 1 | 393.6895 |  |  | 9.321 | 0.7 |
|  |  | 1 | 127.4633 |  |  | 8.786 | 1.792 |
|  |  |  |  |  |  |  |  |
| ELISA | IFNB pg/ml | siNTC 72hpt | siVRK3 72hpt |  |  |  |  |
|  |  | 20.86496 | 474.1583 |  |  |  |  |
|  |  | 11.13509 | 199.5055 |  |  |  |  |
|  |  | 26.2985 | 369.85 |  |  |  |  |
|  |  |  |  |  |  |  |  |
|  | IFNB pg/ml | siNTC 96hpt | siVRK3 96hpt |  |  |  |  |
|  |  | 5.04374 | 800.1603 |  |  |  |  |
|  |  | 137.3483 | 626.8845 |  |  |  |  |
|  |  | 6.1126 | 841.0954 |  |  |  |  |

| **5B** |  | |  | |  | | |  | |  | |  | |  | |  |
| --- | --- | --- | --- | --- | --- | --- | --- | --- | --- | --- | --- | --- | --- | --- | --- | --- |
| RTqPCR | Fold Change | | siNTC 24hpi | | siVRK3 24hpi | | |  | | dCt | | siNTC 24hpi | | siVRK3 24hpi | |  |
|  | IFNB1 | | 1 | | 18.34736 | | |  | | IFNB1 | | 11.50194 | | 7.304436 | |  |
|  |  | | 1 | | 9.563709 | | |  | |  | | 15.06484 | | 11.80727 | |  |
|  |  | | 1 | | 24.8958 | | |  | |  | | 16.51069 | | 11.87286 | |  |
|  |  | |  | |  | | |  | |  | |  | |  | |  |
|  |  | | siNTC 72hpi | | siVRK3 72hpi | | |  | | dCt | | siNTC 72hpi | | siVRK3 72hpi | |  |
|  | IFNB1 | | 1 | | 3.523798 | | |  | | IFNB1 | | 9.173 | | 7.356 | |  |
|  |  | | 1 | | 4.309399 | | |  | |  | | 8.815 | | 6.707 | |  |
|  |  | | 1 | | 9.775893 | | |  | |  | | 6.994 | | 3.704 | |  |
|  |  | |  | |  | | |  | |  | |  | |  | |  |
| ELISA | IFNB pg/ml | | siNTC 24hpi | | siVRK3 24hpi | | |  | |  | |  | |  | |  |
|  |  | | 3.861636 | | 185.8492 | | |  | |  | |  | |  | |  |
|  |  | | 3.939475 | | 217.2962 | | |  | |  | |  | |  | |  |
|  |  | | 24.1405 | | 209.124 | | |  | |  | |  | |  | |  |
|  |  | |  | |  | | |  | |  | |  | |  | |  |
|  | IFNB pg/ml | | siNTC 72hpi | | siVRK3 72hpi | | |  | |  | |  | |  | |  |
|  |  | | 12.2252 | | 210.8202 | | |  | |  | |  | |  | |  |
|  |  | | 3.84683 | | 140.2025 | | |  | |  | |  | |  | |  |
|  |  | | 32.4806 | | 184.304 | | |  | |  | |  | |  | |  |
| **5C** | |  | |  | |  |  | |  | |  | |  | |  | |
| RT2 Profiler Array | | R1 | |  | | R2 |  | | R1 | |  | | R2 | |  | |
|  | | NTC Mock R1 | | VRK3 Mock R1 | | NTC Mock R2 | VRK3 Mock R2 | | NTC 24hpi R1 | | VRK3 24hpi R1 | | NTC 24hpi R2 | | VRK3 24hpi R2 | |
| ADAR | | 0.551231 | | 0.617825 | | 0.164544 | 0.965727 | | 0.526264 | | 0.730472 | | 0.587637 | | 1.622243 | |
| BAG3 | | 1.772438 | | 1.125569 | | 2.931406 | 3.957331 | | 2.369145 | | 1.442249 | | 3.637151 | | 5.320146 | |
| BST2 | | 1.483832 | | 7.170368 | | 2.776422 | 16.93216 | | 1.73944 | | 7.607877 | | 4.277915 | | 24.12175 | |
| CASP1 | | 0.052872 | | 0.354401 | | 0.130532 | 0.297035 | | 0.085601 | | 0.362888 | | 0.13283 | | 0.523176 | |
| CAV1 | | 12.13818 | | 9.508403 | | 17.27178 | 10.18993 | | 15.46136 | | 7.624663 | | 26.43686 | | 17.57644 | |
| CCL2 | | 0.000025 | | 0.000029 | | 0.000117 | 0.000126 | | 0.000026 | | 0.000023 | | 0.00285 | | 0.000078 | |
| CCL5 | | 0.00406 | | 0.140755 | | 0.026398 | 2.441943 | | 0.001686 | | 0.164894 | | 0.030993 | | 0.773616 | |
| CD70 | | 0.00164 | | 0.004774 | | 0.003349 | 0.002131 | | 0.000026 | | 0.001423 | | 0.001402 | | 0.00384 | |
| CD80 | | 0.000025 | | 0.000029 | | 0.000117 | 0.000126 | | 0.000198 | | 0.000041 | | 0.000064 | | 0.000078 | |
| CD86 | | 0.000025 | | 0.000029 | | 0.000117 | 0.000126 | | 0.000026 | | 0.000087 | | 0.005189 | | 0.001869 | |
| CDKN1B | | 0.738062 | | 0.424373 | | 0.350842 | 0.669749 | | 0.85983 | | 0.497494 | | 0.582773 | | 1.166014 | |
| CIITA | | 0.003728 | | 0.001176 | | 0.000614 | 0.001191 | | 0.000586 | | 0.0009 | | 0.000153 | | 0.002133 | |
| CRP | | 0.016495 | | 0.039482 | | 0.058996 | 0.216218 | | 0.019413 | | 0.040043 | | 0.084234 | | 0.283115 | |
| CXCL10 | | 0.000888 | | 0.081279 | | 0.016622 | 1.889177 | | 0.002586 | | 0.088374 | | 0.037011 | | 1.084547 | |
| DDX58 | | 0.783737 | | 4.381591 | | 1.064334 | 8.512895 | | 0.865207 | | 4.234247 | | 1.981911 | | 12.80394 | |
| EIF2AK2 | | 1.312707 | | 2.734522 | | 2.303898 | 4.003589 | | 1.92213 | | 2.534112 | | 4.143787 | | 8.272107 | |
| GBP1 | | 0.436286 | | 2.888048 | | 0.718029 | 2.768337 | | 0.539143 | | 2.251736 | | 1.165029 | | 5.058245 | |
| HLA-A | | 0.763232 | | 3.067428 | | 0.434691 | 2.236083 | | 0.98272 | | 4.113823 | | 0.858629 | | 3.712232 | |
| HLA-B | | 0.000646 | | 0.002466 | | 0.00107 | 0.019905 | | 0.001271 | | 0.006231 | | 0.001583 | | 0.017356 | |
| HLA-E | | 8.017935 | | 16.62619 | | 6.412615 | 15.75181 | | 11.43329 | | 17.19094 | | 13.05566 | | 21.97484 | |
| HLA-G | | 0.001777 | | 0.010851 | | 0.000178 | 0.002849 | | 0.001415 | | 0.003534 | | 0.000145 | | 0.006546 | |
| IFI16 | | 3.206212 | | 4.060127 | | 1.70714 | 5.652401 | | 3.428889 | | 4.260077 | | 4.608962 | | 8.925171 | |
| IFI27 | | 8.260516 | | 11.39164 | | 7.077392 | 45.27723 | | 8.846754 | | 18.60589 | | 14.77288 | | 51.74293 | |
| IFI30 | | 0.043293 | | 0.210208 | | 0.015565 | 0.650241 | | 0.051563 | | 0.21274 | | 0.142355 | | 1.006035 | |
| IFI6 | | 7.000957 | | 37.86854 | | 2.676075 | 50.73825 | | 5.758027 | | 39.74875 | | 8.36912 | | 86.83266 | |
| IFIH1 | | 0.364273 | | 1.83404 | | 0.205536 | 3.741321 | | 0.338102 | | 2.242846 | | 0.531838 | | 4.692247 | |
| IFIT1 | | 0.898572 | | 6.777996 | | 1.093013 | 23.46517 | | 0.811141 | | 7.991458 | | 2.610496 | | 24.57577 | |
| IFIT2 | | 0.000025 | | 1.112566 | | 0.000117 | 3.013506 | | 0.115776 | | 1.026272 | | 0.556755 | | 5.009556 | |
| IFIT3 | | 0.340506 | | 7.801199 | | 0.367441 | 12.04378 | | 0.299457 | | 6.763752 | | 1.275388 | | 15.80134 | |
| IFITM1 | | 1.348378 | | 30.22031 | | 1.748155 | 71.21019 | | 0.99362 | | 37.03534 | | 2.311613 | | 49.68388 | |
| IFITM2 | | 1.720852 | | 1.643555 | | 2.364535 | 4.936898 | | 1.633222 | | 1.997152 | | 3.631617 | | 4.98334 | |
| IFITM3 | | 25.97992 | | 83.70532 | | 78.54104 | 239.6843 | | 32.14844 | | 96.40587 | | 77.54774 | | 152.2614 | |
| IFNA1 | | 0.001291 | | 0.002978 | | 0.000117 | 0.000494 | | 0.003793 | | 0.004833 | | 0.001514 | | 0.001678 | |
| IFNA2 | | 0.000025 | | 0.000029 | | 0.000117 | 0.000126 | | 0.000605 | | 0.000023 | | 0.000064 | | 0.000078 | |
| IFNA4 | | 0.000025 | | 0.000029 | | 0.000117 | 0.000126 | | 0.000026 | | 0.000023 | | 0.000064 | | 0.000078 | |
| IFNAR1 | | 1.138426 | | 0.734233 | | 0.600297 | 1.812174 | | 1.293907 | | 0.826467 | | 1.449554 | | 2.605149 | |
| IFNAR2 | | 0.207745 | | 0.103221 | | 0.1364 | 0.214154 | | 0.226476 | | 0.124514 | | 0.271848 | | 0.228144 | |
| IFNB1 | | 0.002246 | | 0.013788 | | 0.010037 | 0.462127 | | 0.000026 | | 0.022513 | | 0.01501 | | 0.350258 | |
| IFNE | | 0.017237 | | 0.014827 | | 0.015625 | 0.019013 | | 0.026476 | | 0.011115 | | 0.015726 | | 0.044413 | |
| IFNW1 | | 0.000025 | | 0.000653 | | 0.000117 | 0.000126 | | 0.000026 | | 0.000023 | | 0.000064 | | 0.000078 | |
| IL10 | | 0.000025 | | 0.000029 | | 0.000117 | 0.000126 | | 0.00059 | | 0.000023 | | 0.000064 | | 0.000078 | |
| IL15 | | 0.040521 | | 0.075003 | | 0.110239 | 0.129689 | | 0.037722 | | 0.061272 | | 0.067528 | | 0.09655 | |
| IL6 | | 0.020075 | | 0.04085 | | 0.104841 | 0.553453 | | 0.012272 | | 0.053099 | | 0.373616 | | 0.414171 | |
| IRF1 | | 0.174924 | | 0.424088 | | 0.188664 | 0.506691 | | 0.245125 | | 0.388827 | | 0.3547 | | 0.951873 | |
| IRF2 | | 0.359738 | | 0.371129 | | 0.27454 | 0.587045 | | 0.319329 | | 0.378904 | | 0.564013 | | 1.118138 | |
| IRF3 | | 0.883272 | | 0.501106 | | 1.981103 | 2.415823 | | 0.94051 | | 0.651301 | | 2.0648 | | 2.039889 | |
| IRF5 | | 0.001631 | | 0.001833 | | 0.009052 | 0.010438 | | 0.000633 | | 0.001707 | | 0.021893 | | 0.000202 | |
| IRF7 | | 0.111172 | | 0.394602 | | 0.060646 | 0.525585 | | 0.108271 | | 0.360759 | | 0.1516 | | 1.065109 | |
| IRF9 | | 1.441946 | | 1.588043 | | 0.830886 | 2.575559 | | 1.420759 | | 1.617483 | | 1.715776 | | 4.659212 | |
| ISG15 | | 1.676542 | | 13.39153 | | 2.696972 | 57.51857 | | 1.572616 | | 16.92696 | | 3.866448 | | 45.99085 | |
| ISG20 | | 0.997521 | | 3.745978 | | 1.034255 | 7.887077 | | 1.27879 | | 4.403454 | | 1.387509 | | 6.532557 | |
| JAK1 | | 2.438567 | | 2.218617 | | 1.95791 | 2.699671 | | 2.657717 | | 1.990019 | | 4.004663 | | 5.512214 | |
| JAK2 | | 0.088091 | | 0.077902 | | 0.04476 | 0.080556 | | 0.100648 | | 0.080437 | | 0.112983 | | 0.16517 | |
| MAL | | 0.000025 | | 0.000029 | | 0.000117 | 0.000126 | | 0.000026 | | 0.000023 | | 0.000064 | | 0.000078 | |
| MET | | 0.713427 | | 0.830069 | | 1.390579 | 1.930506 | | 0.783001 | | 0.956041 | | 2.23742 | | 2.662194 | |
| MNDA | | 0.000564 | | 0.000037 | | 0.000117 | 0.000126 | | 0.000608 | | 0.004689 | | 0.004946 | | 0.000078 | |
| MX1 | | 1.387957 | | 7.959848 | | 0.505925 | 10.38516 | | 0.839511 | | 8.823783 | | 2.002541 | | 17.77774 | |
| MX2 | | 0.113295 | | 1.942297 | | 0.039257 | 2.75443 | | 0.061204 | | 1.897923 | | 0.106296 | | 4.667351 | |
| MYD88 | | 0.619963 | | 0.737976 | | 0.798262 | 1.983961 | | 0.502335 | | 0.842142 | | 0.993227 | | 1.746457 | |
| NMI | | 1.408887 | | 3.442788 | | 0.98465 | 12.81912 | | 1.186717 | | 4.131739 | | 1.584718 | | 10.74364 | |
| NOS2 | | 0.000025 | | 0.000032 | | 0.000117 | 0.000319 | | 0.000082 | | 0.000091 | | 0.000064 | | 0.000078 | |
| OAS1 | | 1.667536 | | 5.944284 | | 1.578693 | 14.49653 | | 1.51106 | | 6.475673 | | 4.031176 | | 19.6751 | |
| OAS2 | | 2.22854 | | 13.73419 | | 0.992839 | 17.22296 | | 2.069928 | | 15.00756 | | 3.510163 | | 29.59776 | |
| PML | | 1.249291 | | 2.550434 | | 0.969915 | 2.641942 | | 1.534431 | | 2.39003 | | 1.882461 | | 5.491122 | |
| PRKCZ | | 0.000025 | | 0.001107 | | 0.000117 | 0.000126 | | 0.003713 | | 0.00268 | | 0.000064 | | 0.011552 | |
| PSME2 | | 2.200778 | | 3.373977 | | 3.936132 | 8.176196 | | 2.207569 | | 3.373989 | | 5.20598 | | 8.66929 | |
| SH2D1A | | 0.000066 | | 0.000501 | | 0.000117 | 0.006318 | | 0.000026 | | 0.000122 | | 0.000064 | | 0.0001 | |
| SHB | | 0.233521 | | 0.355491 | | 0.376132 | 0.661253 | | 0.361177 | | 0.360856 | | 0.84668 | | 1.097577 | |
| SOCS1 | | 0.008981 | | 0.021718 | | 0.006976 | 0.063138 | | 0.010417 | | 0.026092 | | 0.03182 | | 0.155987 | |
| STAT1 | | 4.284516 | | 9.238308 | | 2.988743 | 16.76599 | | 3.501166 | | 8.669126 | | 6.051643 | | 26.73074 | |
| STAT2 | | 0.754694 | | 1.411548 | | 0.205076 | 1.204311 | | 0.664797 | | 1.38913 | | 0.582819 | | 2.527994 | |
| STAT3 | | 1.729516 | | 1.660363 | | 1.029265 | 3.789133 | | 1.709759 | | 1.73284 | | 2.510123 | | 5.860842 | |
| TAP1 | | 0.946293 | | 3.709612 | | 0.442596 | 5.523362 | | 0.894258 | | 3.641489 | | 1.212915 | | 9.713161 | |
| TICAM1 | | 0.460982 | | 0.260454 | | 0.301417 | 0.84325 | | 0.497914 | | 0.381466 | | 0.682314 | | 1.224998 | |
| TIMP1 | | 2.663487 | | 2.235207 | | 3.269354 | 6.835248 | | 3.071707 | | 2.541218 | | 4.404096 | | 9.325473 | |
| TLR3 | | 0.102976 | | 0.451699 | | 0.065376 | 0.662041 | | 0.096208 | | 0.44835 | | 0.153048 | | 1.160268 | |
| TLR7 | | 0.000041 | | 0.000225 | | 0.000738 | 0.000403 | | 0.000373 | | 0.000143 | | 0.000557 | | 0.000463 | |
| TLR8 | | 0.000025 | | 0.000029 | | 0.000117 | 0.000126 | | 0.000026 | | 0.000023 | | 0.000064 | | 0.000078 | |
| TLR9 | | 0.00054 | | 0.000029 | | 0.000117 | 0.000126 | | 0.000512 | | 0.000531 | | 0.001486 | | 0.000078 | |
| TMEM173 | | 0.418707 | | 0.424874 | | 0.513124 | 0.812912 | | 0.440462 | | 0.438449 | | 0.75688 | | 1.551785 | |
| TNFSF10 | | 0.009499 | | 0.149663 | | 0.016241 | 0.170408 | | 0.01081 | | 0.155529 | | 0.038562 | | 0.339428 | |
| TRAF3 | | 0.794414 | | 0.445214 | | 0.614478 | 1.132636 | | 0.920048 | | 0.47096 | | 1.20827 | | 1.761237 | |
| TYK2 | | 0.57026 | | 0.287956 | | 0.190798 | 0.3614 | | 0.562148 | | 0.302311 | | 0.351392 | | 0.602164 | |
| VEGFA | | 0.20854 | | 0.227635 | | 0.283549 | 1.273613 | | 0.196631 | | 0.26195 | | 0.678643 | | 1.148557 | |

| **5D** |  |  |  |  |  |  |  |
| --- | --- | --- | --- | --- | --- | --- | --- |
| RTqPCR | Fold Change | siNTC 72hpt | siVRK3 72hpt |  | dCt | siNTC 72hpt | siVRK3 72hpt |
|  | OAS1 | 1 | 11.25582 |  | OAS1 | 4.316 | 0.824 |
|  |  | 1 | 7.265754 |  |  | 5.024 | 2.163 |
|  |  | 1 | 7.658497 |  |  | 4.845 | 1.908 |
|  |  | siNTC 72hpt | siVRK3 72hpt |  |  | siNTC 72hpt | siVRK3 72hpt |
|  | OAS2 | 1 | 18.98909 |  | OAS2 | 6.761 | 2.514 |
|  |  | 1 | 15.42931 |  |  | 5.104 | 1.156 |
|  |  | 1 | 8.905717 |  |  | 6.784 | 3.629 |
|  |  | siNTC 72hpt | siVRK3 72hpt |  |  | siNTC 72hpt | siVRK3 72hpt |
|  | IFIT2 | 1 | 60.151 |  | IFIT2 | 7.599 | 1.689 |
|  |  | 1 | 58.06789 |  |  | 6.279 | 3.701 |
|  |  | 1 | 14.72039 |  |  | 8.288 | 4.408 |

| **5E** |  |  |  |  |  |  |  |
| --- | --- | --- | --- | --- | --- | --- | --- |
| RTqPCR | Fold Change | siNTC 24hpi | siVRK3 24hpi |  | dCt | siNTC 24hpi | siVRK3 24hpi |
|  | OAS1 | 1 | 6.070102 |  | OAS1 | 5.034 | 2.433 |
|  |  | 1 | 5.907679 |  |  | 4.252 | 1.689 |
|  |  | 1 | 8.774808 |  |  | 5.645 | 2.512 |
|  |  | siNTC 24hpi | siVRK3 24hpi |  |  | siNTC 24hpi | siVRK3 24hpi |
|  | OAS2 | 1 | 14.77807 |  | OAS2 | 5.584 | 1.699 |
|  |  | 1 | 7.599122 |  |  | 6.428 | 3.502 |
|  |  | 1 | 12.92594 |  |  | 6.187 | 2.495 |
|  |  | siNTC 24hpi | siVRK3 24hpi |  |  | siNTC 24hpi | siVRK3 24hpi |
|  | IFIT2 | 1 | 9.190298 |  | IFIT2 | 7.287 | 4.087 |
|  |  | 1 | 13.3601 |  |  | 7.853 | 4.113 |
|  |  | 1 | 15.26649 |  |  | 8.382 | 4.45 |

| **5F** |  |  |  |
| --- | --- | --- | --- |
| ELISA | IFNB pg/ml | EV 72hpi | VRK3 72hpi |
|  |  | 198.4798 | 165.1792 |
|  |  | 269.9543 | 77.05428 |
|  |  | 200.3421 | 107.9183 |

| **6D** |  |  |  |  |  |
| --- | --- | --- | --- | --- | --- |
| ELISA | IFNB pg/ml | siNTC | siVRK3 | siRIGI | siVRK3+siRIGI |
|  | 96hpt | 13.1293 | 9177.456 | 26.32848 | 2188.933 |
|  |  | 0.3701 | 16221.42 | 21.0488 | 964.4896 |
|  |  | 23.82839 | 17184.96 | 23.2045 | 143.0609 |
|  |  |  |  |  |  |
| ELISA | IFNB pg/ml | siNTC | siVRK3 | siRIGI | siVRK3+siRIGI |
|  | 72hpi | 146.1407 | 426.4032 | 1.250045 | 50.22674 |
|  |  | 213.4565 | 290.0118 | 6.66946 | 8.42935 |
|  |  | 90.70421 | 615.5914 | 4.90957 | 28.22811 |

| **6E** |  |  |  |  |  |
| --- | --- | --- | --- | --- | --- |
| Flow Cytometry | %GFP+ Cells | siNTC | siVRK3 | siRIGI | siVRK3+siRIGI |
|  |  | 36.8 | 25.1 | 44.2 | 39.2 |
|  |  | 32.3 | 15.6 | 32.0 | 37.3 |
|  |  | 31.1 | 18.5 | 34.0 | 33.6 |

| **S1A** |  |  |  |  |  |  |
| --- | --- | --- | --- | --- | --- | --- |
| Flow Cytometry | %RFP+ Cells | siNTC | siVRK3 A | siVRK3 B | siVRK3 C | siVRK3 pool |
|  |  | 16.7 | 14.5 | 11.2 | 10.9 | 10.7 |
|  |  | 18.3 | 13.8 | 10.7 | 10.4 | 7.5 |
|  |  | 19.0 | 12.7 | 6.3 | 6.1 | 3.8 |

| **S1C** |  |  |  |  |  |  |
| --- | --- | --- | --- | --- | --- | --- |
| Flow Cytometry | %RFP+ Cells | siNTC | siVRK3 A | siVRK3 B | siVRK3 C | siVRK3 pool |
|  |  | 15.4 | 10.7 | 5.4 | 0.9 | 3.5 |
|  |  | 18.1 | 9.2 | 3.3 | 2.3 | 4.4 |
|  |  | 23.0 | 11.1 | 9.2 | 4.5 | 6.2 |

| **S2G** |  |  |  |  |  |  |  |
| --- | --- | --- | --- | --- | --- | --- | --- |
| IFA | Index of Correlation | AIF | EEA1 | Lamin B1 | LAMP1 | PDI | RCAS |
|  |  | 0.61 | 0.63 | 0.55 | 0.58 | 0.81 | 0.61 |
|  |  | 0.55 | 0.58 | 0.50 | 0.40 | 0.79 | 0.58 |
|  |  | 0.57 | 0.65 | 0.40 | 0.54 | 0.83 | 0.64 |

| **S3A** |  |  |  |  |
| --- | --- | --- | --- | --- |
| RTqPCR | Fold Change | siNTC | siVRK1 | siVRK3 |
|  | IFNB1 | 1 | 2.022471 | 493.9572 |
|  |  | 1 | 0.654256 | 393.6895 |
|  |  | 1 | 0.722013 | 127.4633 |

| **S3B** |  |  |  |  |
| --- | --- | --- | --- | --- |
| RTqPCR | Fold Change | siNTC | siVRK1 | siVRK3 |
|  | IFNB1 | 1 | 1.450959 | 3.523798 |
|  |  | 1 | 1.815 | 4.309399 |
|  |  | 1 | 1.506493 | 10.848 |

| **S3C** |  |  |  |  |  |  |
| --- | --- | --- | --- | --- | --- | --- |
| ELISA | IFNB pg/ml | siNTC | siVRK3 A | siVRK3 B | siVRK3 C | siVRK3 pool |
|  |  | 171.8146 | 718.402 | 2244.01 | 2505.92 | 1658.934 |
|  |  | 80.5342 | 979.941 | 3408.486 | 2729.493 | 2098.405 |
|  |  | 182.2286 | 426.81 | 2209.834 | 1630.816 | 1768.281 |

| **S3D** |  |  |  |  |  |  |
| --- | --- | --- | --- | --- | --- | --- |
| ELISA | IFNB pg/ml | siNTC | siVRK3 A | siVRK3 B | siVRK3 C | siVRK3 pool |
|  |  | 67.5815 | 154.1108 | 508.132 | 288.4514 | 200 |
|  |  | 80.8425 | 130.752 | 456.637 | 287.41 | 330.1074 |
|  |  | 15.651 | 127.4615 | 296.3885 | 248.9935 | 154.958 |

| **S4B** |  |  |  |
| --- | --- | --- | --- |
| Luciferase Assay | IFNB/Renilla | EV | VRK3 |
|  | cGAS | 0.6747 | 0.5625 |
|  |  | 0.6124 | 0.3556 |
|  |  | 0.6801 | 0.6231 |
|  |  | EV | VRK3 |
|  | STING | 35.4421 | 13.2545 |
|  |  | 38.0208 | 29.9481 |
|  |  | 31.8478 | 22.8682 |
|  |  | EV | VRK3 |
|  | cGAS+STING | 32.1589 | 27.1084 |
|  |  | 59.0392 | 32.3021 |
|  |  | 45.0010 | 12.7322 |
|  |  | EV | VRK3 |
|  | TBK1 | 237.485 | 140.9203 |
|  |  | 301.7973 | 245.4284 |
|  |  | 400.5235 | 361.5428 |
|  |  | EV | VRK3 |
|  | IRF3 | 0.8823 | 0.7436 |
|  |  | 0.9481 | 0.8445 |
|  |  | 0.7543 | 0.6750 |

| **S4C** |  |  |  |  |
| --- | --- | --- | --- | --- |
| ELISA | 2'3'-cGAMP pg/ml | siNTC | siVRK3 | siBANF1 |
|  |  | 281.03 | 200.326 | 465.763 |
|  |  | 234.5655 | 251.7885 | 433.866 |
|  |  | 406.5455 | 320.4415 | 560.844 |

| **S5A** |  |  |  |  |  |
| --- | --- | --- | --- | --- | --- |
| Flow Cytometry | %GFP+ Cells | siNTC | | siVRK3 | |
|  |  | Isotype Ab | IFNB Ab | Isotype Ab | IFNB Ab |
|  |  | 42.4 | 47.4 | 30.2 | 41.4 |
|  |  | 38.9 | 40.9 | 33.5 | 35.7 |

| **S5B** |  |  |  |  |  |
| --- | --- | --- | --- | --- | --- |
| Flow Cytometry | %GFP+ Cells | siNTC | siVRK3 | siIFNB1 | siVRK3+siIFNB1 |
|  |  | 41.1 | 28.5 | 43.4 | 44.9 |
|  |  | 35.7 | 17.71 | 35.5 | 30.6 |
|  |  | 31.4 | 23.2 | 40.6 | 38.3 |
